# Callus γδ T cells and microbe-induced intestinal Th17 cells improve fracture healing in mice

**DOI:** 10.1172/JCI166577

**Published:** 2023-04-17

**Authors:** Hamid Y. Dar, Daniel S. Perrien, Subhashis Pal, Andreea Stoica, Sasidhar Uppuganti, Jeffry S. Nyman, Rheinallt M. Jones, M. Neale Weitzmann, Roberto Pacifici

**Affiliations:** 1 Division of Endocrinology, Metabolism and Lipids, Department of Medicine and; 2 Emory Microbiome Research Center, Emory University, Atlanta, Georgia, USA.; 3 Department of Orthopedic Surgery, Vanderbilt University Medical Center, Nashville, Tennessee, USA.; 4 Department of Veterans Affairs, Tennessee Valley Healthcare System, Nashville, Tennessee, USA.; 5 Division of Pediatric Gastroenterology, Hepatology, and Nutrition, Department of Pediatrics, Emory University, Atlanta, Georgia, USA.; 6 Atlanta VA Health Care System, Department of Veterans Affairs, Decatur, Georgia, USA.; 7 Immunology and Molecular Pathogenesis Program, Emory University, Atlanta, Georgia, USA.

**Keywords:** Bone Biology, Microbiology, Bone disease, Orthopedics, T cells

## Abstract

IL-17A (IL-17), a driver of the inflammatory phase of fracture repair, is produced locally by several cell lineages including γδ T cells and Th17 cells. However, the origin of these T cells and their relevance for fracture repair are unknown. Here, we show that fractures rapidly expanded callus γδ T cells, which led to increased gut permeability by promoting systemic inflammation. When the microbiota contained the Th17 cell–inducing taxon segmented filamentous bacteria (SFB), activation of γδ T cells was followed by expansion of intestinal Th17 cells, their migration to the callus, and improved fracture repair. Mechanistically, fractures increased the S1P receptor 1–mediated (S1PR1-mediated) egress of Th17 cells from the intestine and enhanced their homing to the callus through a CCL20-mediated mechanism. Fracture repair was impaired by deletion of γδ T cells, depletion of the microbiome by antibiotics (Abx), blockade of Th17 cell egress from the gut, or Ab neutralization of Th17 cell influx into the callus. These findings demonstrate the relevance of the microbiome and T cell trafficking for fracture repair. Modifications of microbiome composition via Th17 cell–inducing bacteriotherapy and avoidance of broad-spectrum Abx may represent novel therapeutic strategies to optimize fracture healing.

## Graphical abstract

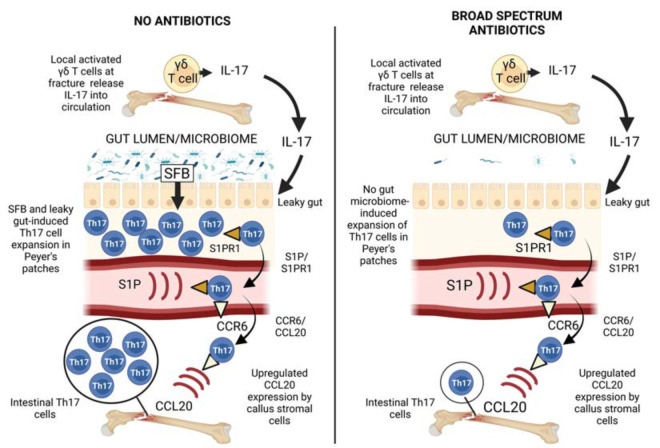

## Introduction

Fracture healing is a complex process involving several sequential phases (1). At first, blood vessels near the injury rupture to form a hematoma, which is rapidly infiltrated by immune cells (1) that release cytokines, eliciting local and systemic inflammation (2). The inflammatory local microenvironment facilitates the removal of debris and activates chemokines that recruit mesenchymal progenitor cells to resolve inflammation and initiate the reparative phase (3, 4).

IL-17 is a cytokine expressed at high levels immediately after bone injuries. It promotes bone regeneration by increasing the number of osteoprogenitor cells in the early phase of bone fracture healing (5, 6). In addition, IL-17 induces the secretion of other factors involved in the fracture repair process, including TNF-α (TNF), IL-1, and IL-6 (7–9). In a study using the drill-hole injury model, healing was impaired in mice lacking IL-17 (6), a finding that supports its relevance in fracture healing. However, the role of IL-17 remains to be determined in fractures that are repaired by endochondral bone formation, a process in which cartilage is formed within the bone defect and then replaced by bone.

A source of IL-17 is γδ T cells, although the exact role of these cells in fracture repair remains to be defined, as both beneficial and unfavorable effects have been reported (6, 10). The origin of γδ T cells relevant for fracture healing is also unknown. γδ T cells reside in musculoskeletal tissues, where they are quickly activated by damage-associated molecules and stress-induced self-antigens released in the fractured area (6). However, γδ T cells are also abundant in the intestinal epithelial tissues, where they contribute to the maintenance of epithelial barrier integrity (11, 12). Intestinal γδ T cells are activated and polarized by the gut microbiome (13, 14) and have the capacity to migrate to the lamina propria (15). It is presently unknown whether intestinal γδ T cells migrate to the callus to contribute to the inflammatory phase of fracture repair.

Th17 cells, a lineage of CD4^+^ T cells defined by their capacity to release IL-17, are the major source of IL-17 during inflammation (16–18). However, the contribution of Th17 cells to fracture repair remains unknown. In the mouse, Th17 cells are mostly produced in the intestinal lamina propria in response to specific elements of the microbiota (19–21). In healthy mice, differentiation of intestinal Th17 cells may be induced by segmented filamentous bacteria (SFB) (19), which are a spore-forming, Gram-positive commensal microbe (20, 21). In addition, infections with extracellular pathogens such as *Candida albicans* and *Citrobacter rodentium* also activate Th17 cell expansion in the gut tissue. In humans, approximately 20 nonvirulent gut bacterial strains are known to induce Th17 cell differentiation, but their frequency in healthy individuals is variable (22, 23). Animal and human studies have shown that inflammatory conditions such as fractures increase gut permeability (24–28), which in turn leads to increased bacterial translocation across the intestinal epithelium and T cell activation (29, 30). Increased gut permeability further facilitates the expansion of intestinal Th17 cells (31) and γδ T cells (13, 14). Th17 cells express the sphingosine 1 phosphate (S1P) receptor 1 (S1PR1) and egress from gut-associated lymphoid tissues when attracted to the circulating S1PR1 ligand S1P (32). Once in the circulation, Th17 cells home to distant peripheral sites of inflammation. For example, intestinal Th17 cells migrate to the bone marrow (BM) in response to ovariectomy (31) and parathyroid hormone (PTH) (33). Similarly, studies in autoimmune disorders have shown that Th17 cells migrate from the gut to the lungs, kidneys, and joints (34–36). The homing of Th17 cells to inflamed peripheral areas is driven by CCL20, a ligand for the Th17 cell receptor CCR6, which is induced by TNF at sites of inflammation (37–39).

It is presently unknown whether Th17 cells represent a source of IL-17 essential for fracture repair. Also unknown is whether the Th17 cells involved in fracture repair originate at the fracture site, or if they are first produced in the gut and then home to the callus. Here, we examined the role of the gut microbiota and gut permeability in the generation of intestinal γδ T cells and Th17 cells, their homing to the callus, and their role in fracture repair. We show that fractures first activated callus γδ T cells. Although some callus γδ T cells originated in the gut, fractures did not increase the homing of intestinal γδ T cells to the BM. The systemic inflammation triggered by fracture-induced increased gut permeability led to intestinal Th17 cell expansion through a microbiome-dependent process, followed by their migration to the callus along a chemokine gradient induced by callus inflammation. Moreover, we show that blockade of Th17 cell egress from the gut, or blockade of Th17 cell influx into the callus, prevented the expansion of callus Th17 cells and impaired fracture repair. Together, we demonstrated that IL-17 produced by γδ T cells and intestinal Th17 cells played a pivotal role in optimizing fracture repair.

## Results

### SFB-containing microbiome potentiates the production of callus and intestinal cytokines induced by fractures.

To determine the contribution of the Th17 cell–inducing bacteria SFB to the inflammatory stage of fracture repair, female C57BL/6 mice lacking SFB were purchased from The Jackson Laboratory (hereafter referred to as SFB^–^ JAX mice). We then generated SFB^+^ JAX mice by oral gavaging SFB^–^ JAX mice with a liquid suspension of fecal pellets collected from SFB monocolonized mice as previously described (31, 33). SFB^+^ mice develop high numbers of intestinal Th17 cells, whereas SFB^–^ mice harbor a paucity of gut Th17 cells (31, 33). A subset of mice were treated with absorbable broad-spectrum antibiotics (Abx) (1 mg/mL ampicillin, 0.5 mg/mL vancomycin, 1 mg/mL neomycin sulfate, and 1 mg/mL metronidazole dissolved in water) for the duration of the experiment, starting 7 days before fracture induction to ablate the gut microbiota (31, 33). Control mice were treated with sterile water (no Abx). Abx treatment eliminated greater than 99% of the intestinal microbiome in both SFB^+^ and SFB^–^ mice (Supplemental Figure 1A; supplemental material available online with this article; https://doi.org/10.1172/JCI166577DS1), confirming the results of earlier reports (33, 40).

**Figure 1:**
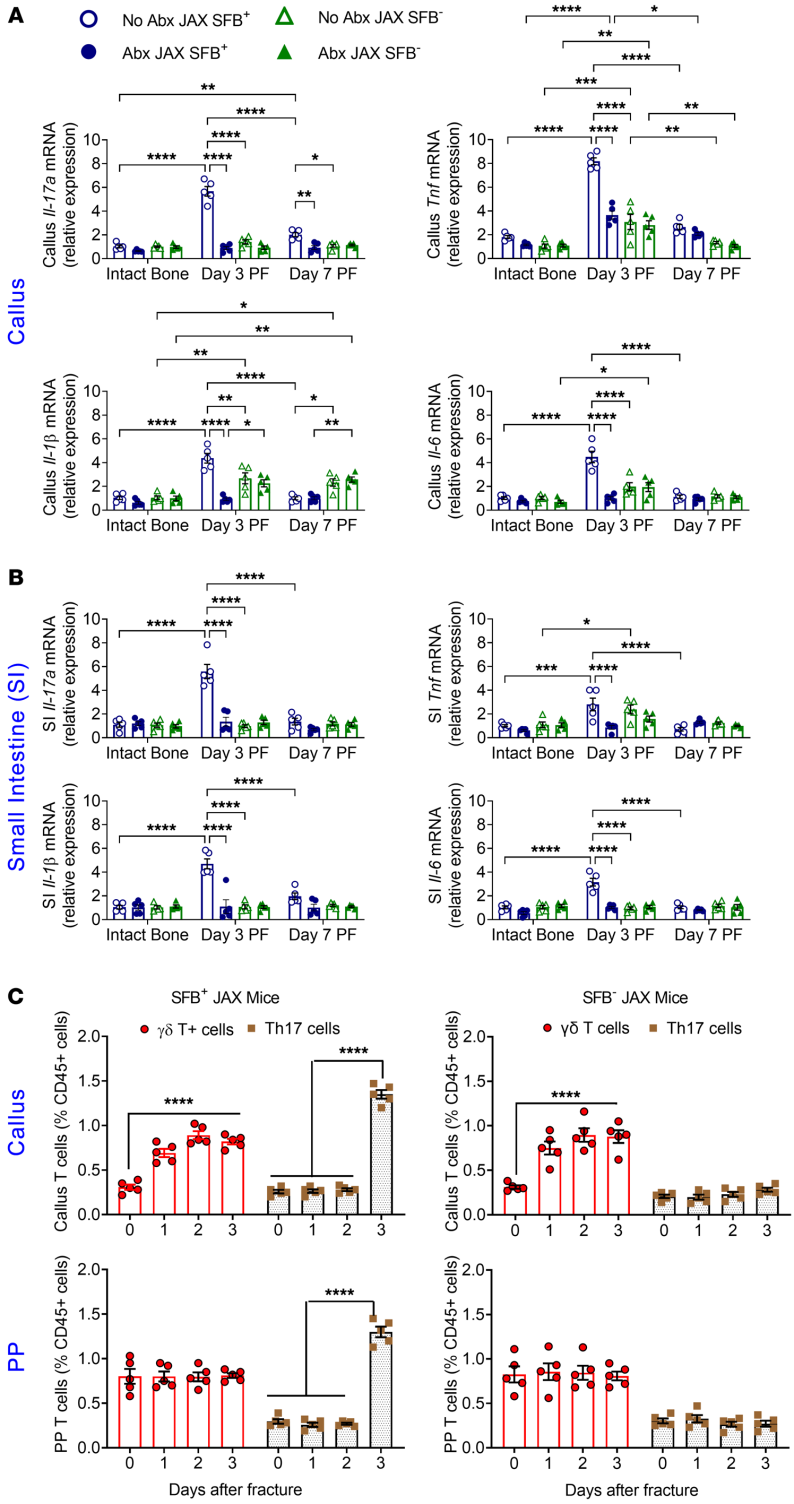
Effects of fractures and Abx-induced microbiota depletion on callus and intestinal inflammatory cytokine transcripts and on the relative number of callus and intestinal γδ T cells and Th17 cells. (**A**) Effects of fractures on the levels of *Il17a*, *Tnf*, *Il1b*, and *Il6* transcripts in the callus of SFB^+^ and SFB^–^ JAX mice. (**B**) Effects of fractures on the levels of *Il17a*, *Tnf*, *Il1b*, and *Il6* transcripts in the SI of SFB^+^ and SFB^–^ JAX mice. (**C**) Effects of fractures on the relative frequency of γδ T cells (CD3ε^+^CD45^+^TCRγδ^+^) and Th17 cells (TCRβ^+^CD45^+^CD4^+^IL-17A^+^) in the callus and PPs of SFB^+^ and SFB^–^ JAX mice. Femoral fractures were induced in 12-week-old female SFB^+^ JAX and SFB^–^ JAX mice. Mice were treated or not with broad-spectrum Abx starting 1 week before fracture surgery. PP and callus cells were recovered daily for 3 days after fracture surgery and analyzed by flow cytometry. Time 0 indicates intact bone. *n* = 5 mice/group. Data are expressed as the mean ± SEM. All data were normally distributed according to the Shapiro-Wilk normality test and analyzed by 2-way ANOVA with post hoc Bonferroni correction for multiple comparisons. *P < 0.05, **P < 0.01, ***P < 0.001, and ****P < 0.0001 compared with the indicated group. Nonsignificant comparisons are not shown.

Femoral fractures were induced by 3-point bending of the femur in 12-week-old female mice. Stabilization of the fracture was achieved by inserting an intramedullary pin prior to fracture to maintain axial alignment during the fracture and avoid large displacements. We then measured cytokine transcript levels in excised callus tissue up to 7 days post fracture (PF). In non–Abx-treated SFB^+^ JAX mice, we found increased levels of *Il17a*, *Tnf*, *Il1b*, and *Il6* transcripts on day 3 PF (Figure 1A). Abx treatment blunted the fracture-induced increase in all measured cytokine transcripts, indicating that the microbiome increased the early inflammatory response to fractures. By contrast, in SFB^–^ mice, the fractures caused a smaller increase in *Tnf* and *Il1b* expression on day 3 that was not blunted by Abx (Figure 1A), suggesting that fractures alter callus cytokines, in large part, via an SFB-dependent mechanism. These data implied that the presence of gut microbiome populations capable of inducing Th17 cells enhanced the production of inflammatory cytokines in the fracture callus. The changes in callus cytokine levels induced by fractures were mirrored by similar changes in cytokine expression in the small intestine (SI). In SFB^+^ mice, the fractures were followed by increased SI expression of *Il17a*, *Tnf*, *Il1b*, and *Il6* transcripts at day 3 PF, which was blocked by Abx (Figure 1B). In SFB^–^ mice, the fractures increased the expression levels of SI *Tnf* transcripts only, and such increase was not prevented by Abx (Figure 1B).

We purchased SFB^+^ mice from Taconic (hereafter referred to as TAC mice) to investigate the effects of Abx in an additional line of SFB^+^ mice and to confirm that the effects of absorbable Abx on cytokine expression arose from their direct effects on the intestinal bacteria, rather than from off-target effects of the Abx. TAC mice were treated with a reduced cocktail of 2 nonabsorbable Abx (2 mg/mL neomycin sulfate, 2 mg/mL bacitracin dissolved in drinking water) for the duration of the experiment, starting 1 week before fracture induction. Analysis of fecal bacterium–specific DNA revealed that nonabsorbable Abx were as effective as broad-spectrum Abx, as they ablated the gut microbiota by more than 99% compared with controls (Supplemental Figure 1B). Mice were subjected to fractures, and cytokine transcript levels were measured in the callus and SI at days 3 and 7 PF. In non–Abx-treated TAC SFB^+^ control mice, callus and SI *Il17a*, *Tnf*, *Il1b*, and *Il6* transcript expression levels increased at day 3 PF (Supplemental Figure 2, A and B). Treatment with nonabsorbable Abx blunted the fracture-induced increase in all measured cytokine transcript levels. Since nonabsorbable Abx and broad-spectrum Abx were both effective in altering cytokine expression, it is unlikely that the activity of Abx was due to an off-target effect of these agents.

Further analysis of intracellular cytokine production by flow cytometry revealed that fractures increased the cellular production of IL-17, TNF, IL-1β, and IL-6 proteins in PPs and callus cells in SFB^+^ mice, but not in SFB^–^ mice at day 3 PF (Supplemental Figure 3, A–D).

### Fractures induced a microbe-independent expansion of γδ T cells and a microbiome-dependent expansion of Th17 cells.

To investigate the contribution of T cells to the fracture-induced production of inflammatory cytokines, cells were collected from the callus tissue and PPs (as opposed to the lamina propria because of the higher T cell yield per mouse in PPs) and analyzed by flow cytometry. These studies revealed that fractures caused an increase in callus γδ T cells (CD3ε^+^CD45^+^TCRγδ^+^ cells) that began on day 1 PF. The expansion of callus γδ T cells occurred in both SFB^+^ and SFB^–^ mice and was not associated with changes in the frequency of PP γδ T cells (Figure 1C). The expansion of callus γδ T cells was followed by an increase in PP and callus Th17 cells (TCRβ^+^CD45^+^CD4^+^IL-17A^+^ cells). The increase in these cells occurred on day 3 PF and took place only in SFB^+^ mice (Figure 1C). These findings are in keeping with the notion that γδ T cells expand locally in response to tissue damage and contribute to the very early stage of fracture repair, whereas callus Th17 cells originate at distant sites and then migrate to the callus.

**Figure 2:**
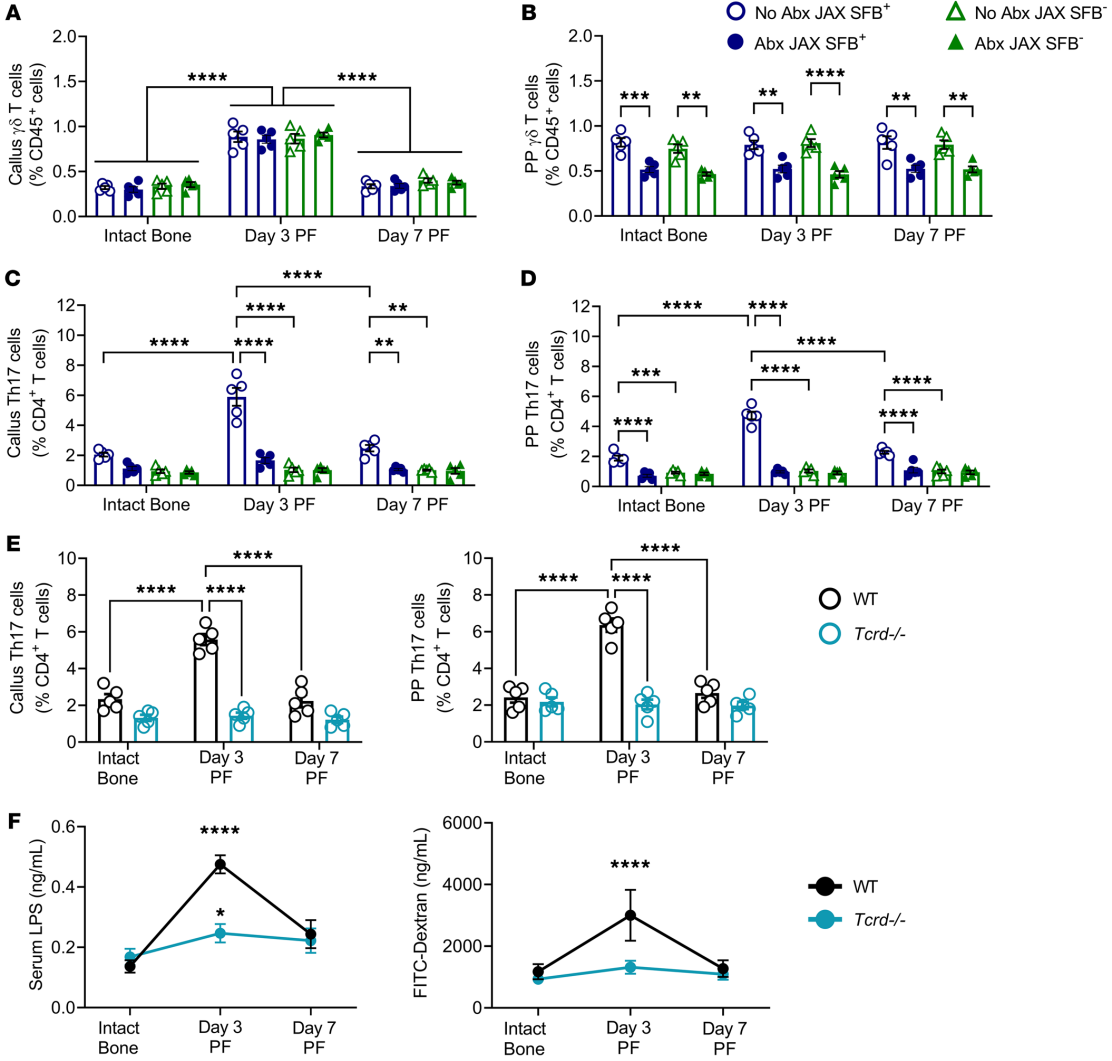
Effects of fractures and Abx-induced microbiota depletion on callus and intestinal γδ T cells and Th17 cells, and gut permeability in WT and γδ T cell^–/–^ mice. Femoral fractures were induced in 12-week-old female SFB^+^ JAX mice, SFB^–^ JAX mice, and SFB^+^ T*crd^–/–^* mice, a strain lacking γδ T cells. SFB^+^ JAX mice and SFB^–^ JAX mice were treated or not with broad-spectrum Abx starting 1 week before fracture surgery. Frequencies of (**A**) callus γδ T cells (CD3ε^+^CD45^+^TCRγδ^+^ cells), (**B**) PP γδ T cells, (**C**) callus Th17 cells (TCRβ^+^CD45^+^CD4^+^IL-17A^+^ cells), and (**D**) PP Th17 cells. (**E**) Callus and PP Th17 cells in T*crd^–/–^* mice. (**F**) Gut permeability in SFB^+^ WT mice and SFB^+^ T*crd^–/–^* mice. Gut permeability was assessed by serum LPS levels and FITC-dextran absorption. *n* = 5 mice/group. Data are expressed as the mean ± SEM. All data were normally distributed according to the Shapiro-Wilk normality test and analyzed by 2-way ANOVA and with post hoc Bonferroni correction for multiple comparisons. **P < 0.01, ***P < 0.001, and ****P < 0.0001 compared with the indicated group (**A**–**E**) or compared with intact bone (**F**). Nonsignificant comparisons are not shown.

Further studies confirmed that fractures increased γδ T cells in the callus but not in the PPs of SFB^+^ and SFB^–^ mice (Figure 2, A and B). Moreover, Abx decreased the frequency of PP γδ T cells (Figure 2B), but they did not alter the changes in callus γδ T cells induced by fractures (Figure 2A). These findings showed that the differentiation of intestinal γδ T cells was, at least in part, microbiota dependent, whereas the frequency of callus γδ T cells was microbiome independent. Together, these data suggest that most of the callus γδ T cells did not originate in the gut. Moreover, in SFB^+^ mice, fractures were followed by an increase in callus and PP Th17 cells, which peaked at day 3 PF and was blunted by Abx treatment (Figure 2, C and D). By contrast, in SFB^–^ mice, fractures were not followed by an expansion of PP or callus Th17 cells (Figure 2, C and D). Further analysis of PP and BM Th17 cells (CD45^+^CD3^+^CD4^+^IL-17A^+^ cells) revealed that fractures were followed by a relative increase in callus and PP effector memory (CD44^+^CD62L^–^CD127^+^), tissue-resident memory (CD44^+^CD62L^–^CD69^+^), central memory (CD44^+^CD62L^–^CD127^+^), and effector (CD44^+^CD62L^–^CD127^–^) Th17 cells, an increase that peaked on day 3 PF (Supplemental Figures 4 and 5**)**. Together, these data indicate that an SFB-containing microbiome is required for fractures to expand intestinal and callus Th17 cells.

To further investigate the temporal relationship between γδ T cells and Th17 cells, we conducted studies in SFB^+^ *Tcrd^–/–^* mice and SFB^+^ WT control mice. *Tcrd^–/–^* mice are completely void of γδ T cells. In these mice, the fractures failed to expand the frequencies of callus and PP Th17 cells (Figure 2E), indicating that γδT cells were required for the subsequent expansion of Th17 cells. Activation and expansion of γδ T cells in the callus and the resulting γδ T cell production of IL-17 is a pivotal driver of systemic inflammation in the early stages of fracture repair (10). Furthermore, one consequence of systemic inflammation is increased gut permeability (24–27). Accordingly, measurements of serum LPS and FITC-dextran absorption — 2 established markers of gut permeability (41, 42) — revealed that fractures increased gut permeability, with a peak at day 3 PF in WT mice (Figure 2F). Confirming the pivotal role of γδ T cells in inducing a leaky gut phenotype, fractures failed to increase gut permeability in *Tcrd^–/–^* mice (Figure 2F). In WT mice, gut permeability peaked at a time corresponding to the maximal expansion of intestinal Th17 cells, a finding confirming an earlier report that increased gut permeability potentiates the capacity of SFB to induce Th17 cell production (31).

We conducted confirmatory studies treating SFB^+^ TAC mice with nonabsorbable Abx. In non–Abx-treated controls, fractures had increased γδ T cells at day 3 in callus but not in PPs (Supplemental Figure 2C). Although nonabsorbable Abx decreased the frequency of PP γδ T cells, they did not alter the changes in callus γδ T cells induced by fractures (Supplemental Figure 2C). Moreover, fractures were followed by an increase in callus and PP Th17 cells at day 3 PF, which was blunted by treatment with nonabsorbable Abx (Supplemental Figure 2D). Together, these findings indicate that the capacity of Abx to affect the frequency of γδ T cells and Th17 cells was not a result of off-target effects of these agents.

### Fractures increase the trafficking of Th17 cells from the SI to the callus.

SFB is known to induce Th17 cells with focused antigenic specificity. As a result, most of the Th17 cells produced in the lamina propria of SFB^+^ mice contain the Vβ14 chain in their TCR receptor (43). Flow cytometric analysis revealed that fractures increased the number of Vβ14^+^Th17 cells in callus tissue of SFB^+^ mice, an effect that was abrogated by broad-spectrum Abx (Supplemental Figure 6A). By contrast, fractures failed to expand Vβ14^+^Th17 cells in callus tissue of SFB^–^ mice (Supplemental Figure 6B). The finding of Th17 cells of intestinal origin in the callus supports the hypothesis that Th17 cells migrate from the gut to the callus. To directly investigate the effect of fractures on T cell trafficking, we made use of C57BL/6 Kaede mice (44). This strain offers a sensitive means to track the migration from the gut to anatomically distant sites of any leukocyte cell type definable by surface-displayed or intracellular markers. Kaede mice ubiquitously express the photoconvertible protein Kaede, which permanently changes its fluorescence emission from green (518 nm) to red (582 nm) upon photoactivation with near-UV light (350–410 nm). Once photoconverted in the intestine, red fluorescing cells can be detected and enumerated by flow cytometry in other organs. The photoconversion of intracellular Kaede has no effect on cellular function or on the homing capacity of T cells (45). Hereafter, we will refer to photoconverted cells as KaedeR cells. The original colony of Kaede mice established in our vivarium was SFB^–^. To utilize a model in which Th17 cells can expand in the gut, we generated SFB^+^ Kaede mice by gavaging the mice with a liquid suspension of stools from mice previously monocolonized with SFB, using established methods (33).

**Figure 3:**
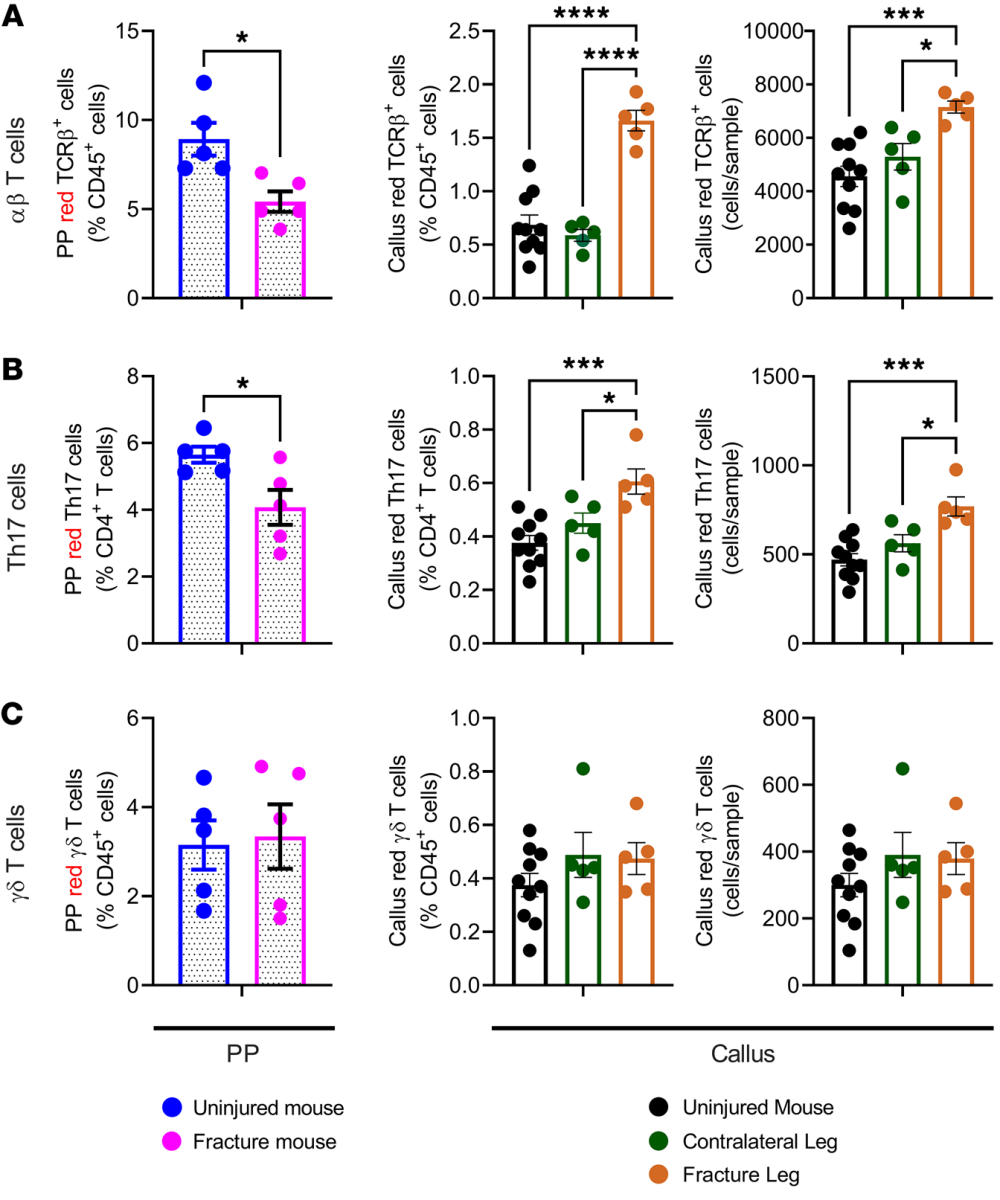
Fractures increase homing to the callus of intestinal αβ T cells and Th17 cells but not γδ T cells. Femoral fractures were induced in 12-week-old male SFB^+^ Kaede mice. After 2 days, 4 PPs were surgically exposed and illuminated with a near-UV light for 2 minutes. After 1 day, mice were sacrificed, and the frequency of PPs and callus red-fluorescing αβ T cells, Th17 cells, and γδ T cells was determined by flow cytometry. PP red-fluorescing T cells were counted in PPs from mice with fractures and PPs from uninjured control mice. Callus red-fluorescing T cells were counted in the callus tissue of fractured femurs, BM from the contralateral uninjured femur, and BM from uninjured mice. (**A**) Relative frequency of PP αβ T cells and relative and absolute frequency of callus αβ T cells. (**B**) Relative frequency of PP Th17 cells and relative and absolute frequency of callus Th17 cells. (**C**) Relative frequency of PP γδ T cells and relative and absolute frequency of callus γδ T cells. *n* = 6 mice/group. Data are expressed as the mean ± SEM. All data were normally distributed according to the Shapiro-Wilk normality test and analyzed by 2-way ANOVA with post hoc Bonferroni correction for multiple comparisons (callus panels), or by unpaired, 2-tailed *t* test (PP panels). *P < 0.05, ***P < 0.001, and ****P < 0.0001 compared with the indicated group. Nonsignificant comparisons are not shown.

To study the effects of fractures on intestinal T cell migration to the callus, closed fractures of the femur were induced in 12-week-old male SFB^+^ Kaede mice. Two days later, 4 PPs were surgically exposed and photoconverted by exposure to a 390 nm light for 2 minutes each. To make sure that no other cells were photoconverted, the whole mouse was covered with aluminum foil. Twenty-four hours later, PP and callus T cells were recovered, and the frequency of KaedeR αβ T cells, Th17 cells, and γδ T cells was determined by flow cytometry, using methods established in previous studies (31). Since only photoconverted intestinal cells fluoresce red, this method provides a measure of the number of intestinal T cells that migrated to the callus in the previous 24 hours. The fraction of KaedeR T cells in the callus was low because only 4 PPs were photoconverted, whereas T cells from all PPs and other intestinal lymphoid tissues migrate to the callus. Since calculations of the absolute number of PP cells were inaccurate because of the variable size of the collected PP tissue, PP KaedeR T cells were quantified only as a percentage of total cells. Fractures decreased the numbers of KaedeR αβ T cells and Th17 cells in PPs (Figure 3, A and B, and Supplemental Figure 7). By contrast, fractures increased the frequency of these cell lineages in the callus (Figure 3, A and B, and Supplemental Figure 7), demonstrating that fractures were followed by increased homing of intestinal αβ T cells and Th17 cells to the callus tissue. Twenty-four hours after photoactivation, we found KaedeR γδ T cells in PPs and BM of intact control mice. However, fractures did not affect the frequency of KaedeR γδ T cells in either PPs or the callus (Figure 3C). Thus, while some intestinal γδ T cells migrated from the gut to the BM, fractures did not increase the migration of γδ T cells to the callus.

**Figure 4:**
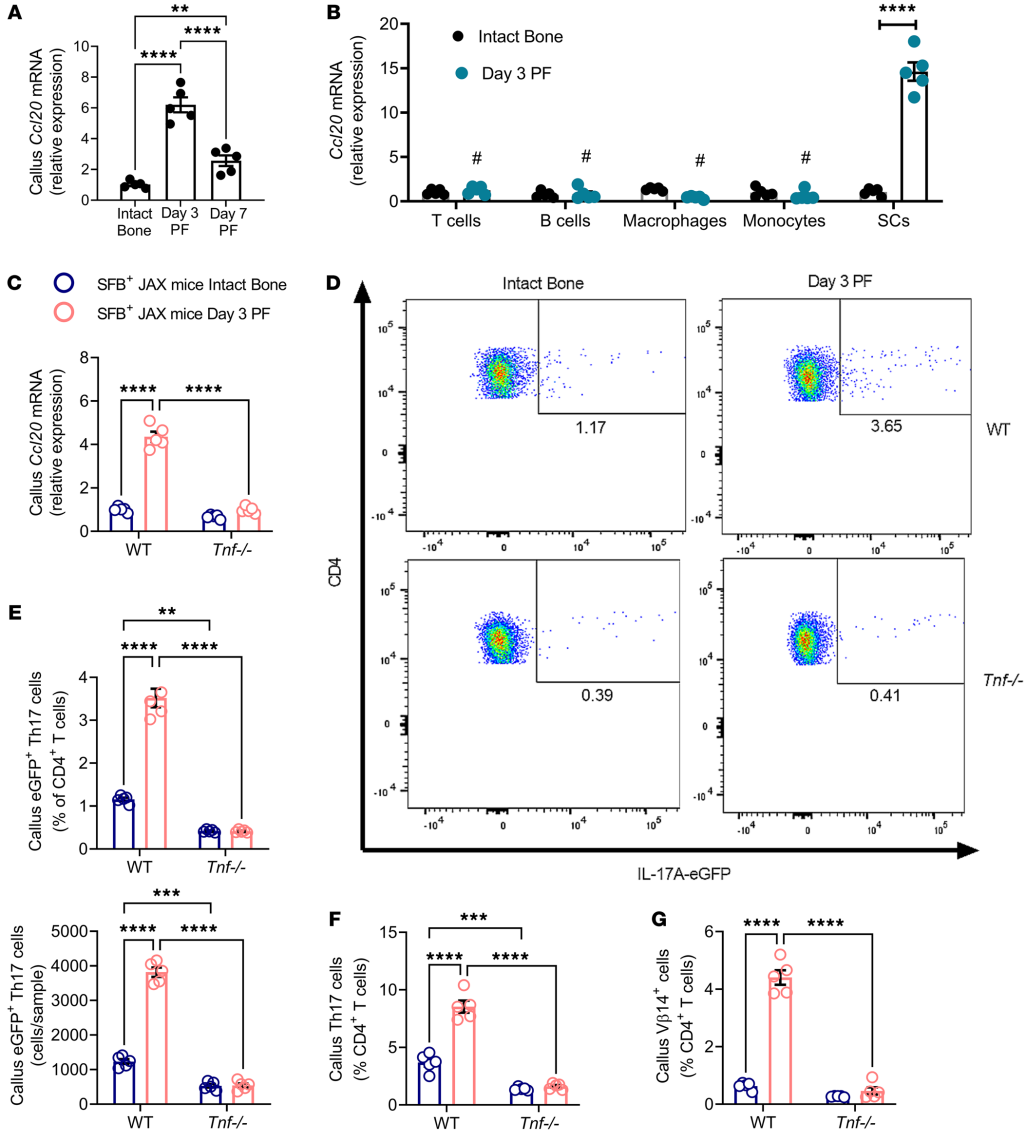
Fractures increase the tropism of Th17 cells to the callus via a TNF-dependent mechanism. (**A**) Callus *Ccl20* transcript levels at days 3 and 7 PF in SFB^+^ TAC mice. (**B**) *Ccl20* transcript levels in purified callus cells at day 3 PF. (**C**) Callus *Ccl20* transcript levels in SFB^+^ WT and *Tnf^–/–^* mice at day 3 PF. (**D** and **E**) Relative and absolute frequencies of EGFP^+^ Th17 cells in the callus of SFB^+^ WT and *Tnf^–/–^* mice subjected to fracture 2 days before adoptive transfer of IL-17A-EGFP^+^ cells. (**F**) Relative and absolute frequencies of callus Th17 cells in SFB^+^ WT mice and *Tnf^–/–^* mice. (**G**) Relative frequency of callus Vβ14^+^ Th17 cells in SFB^+^ WT and *Tnf^–/–^* mice. *n* = 5–6 mice/group. Data are expressed as the mean ± SEM. All data were normally distributed according to the Shapiro-Wilk normality test and analyzed by 2-way ANOVA with post hoc Bonferroni correction for multiple comparisons. **P < 0.01, ***P < 0.001, and ****P < 0.0001 compared with the indicated group. ^#^*P* < 0.001 compared with day 3 PF SCs. Nonsignificant comparisons are not shown.

Homing of Th17 cells to the area of inflammation is driven by the chemokine ligand CCL20, which is expressed by cells at sites of inflammation (37–39), and by the chemokine receptor CCR6, which is expressed by Th17 cells (46), suggesting that CCL20/CCR6 signaling may drive the homing of intestinal Th17 cell to the callus. In support of this hypothesis, we found that fractures increased the transcript levels of CCL20 in callus cells of SFB^+^ mice with a peak at day 3 (Figure 4A). Analysis of purified cells revealed that fractures upregulated the production of CCL20 by stromal cells (SCs) in the callus (Figure 4B). Since CCL20 is induced by TNF (31, 33), we investigated the hypothesis that callus TNF might be required for the homing of Th17 cells to the callus. In support of this hypothesis, we found that fractures upregulated transcript levels of callus CCL20 in SFB^+^ Jax WT mice but not in SFB^+^ JAX *Tnf^–/–^* mice (Figure 4C). To further explore the role of TNF in the homing of intestinal Th17 cells to the callus, we used *Il17a*-EGFP mice, a strain possessing an IRES-EGFP sequence after the stop codon of the *Il17a* gene so that EGFP expression is limited to IL-17A–expressing cells, thus allowing Th17 cells to be detected by measuring EGFP by flow cytometry. Splenic naive CD4^+^ cells (CD4^+^CD44^lo^CD62L^hi^ cells), which are EGFP^–^, were purified from *Il17a*-EGFP mice and cultured in Th17 cell–polarizing conditions for 4 days. Th17 cells (CD4^+^EGFP^+^ cells) were then FACS sorted and transferred into WT and *Tnf^–/–^* mice that had been subjected to fractures 2 days earlier. One day after T cell transfer, the recipient mice were sacrificed and callus Th17 cells (CD4^+^EGFP^+^ cells) enumerated by flow cytometry. WT mice subjected to fractures had a higher relative and absolute number of EGFP^+^ Th17 cells in the callus as compared with nonfractured WT controls (Figure 4, D and E). By contrast, fractures did not increase the frequency of callus EGFP^+^ Th17 cells in *Tnf^–/–^* mice (Figure 4, D and E). Moreover, fractures upregulated the frequency of callus Th17 cells and callus Vβ14^+^ Th17 cells in WT mice but not in *Tnf^–/–^* mice (Figure 4, F and G). These findings and data showing increased callus levels of TNF (Figure 1A) demonstrated that fractures increased the recruitment of intestinal Th17 cells to the callus via a TNF-dependent mechanism.

### γδ T cells and Th17 cells improve fracture healing.

**Figure 5:**
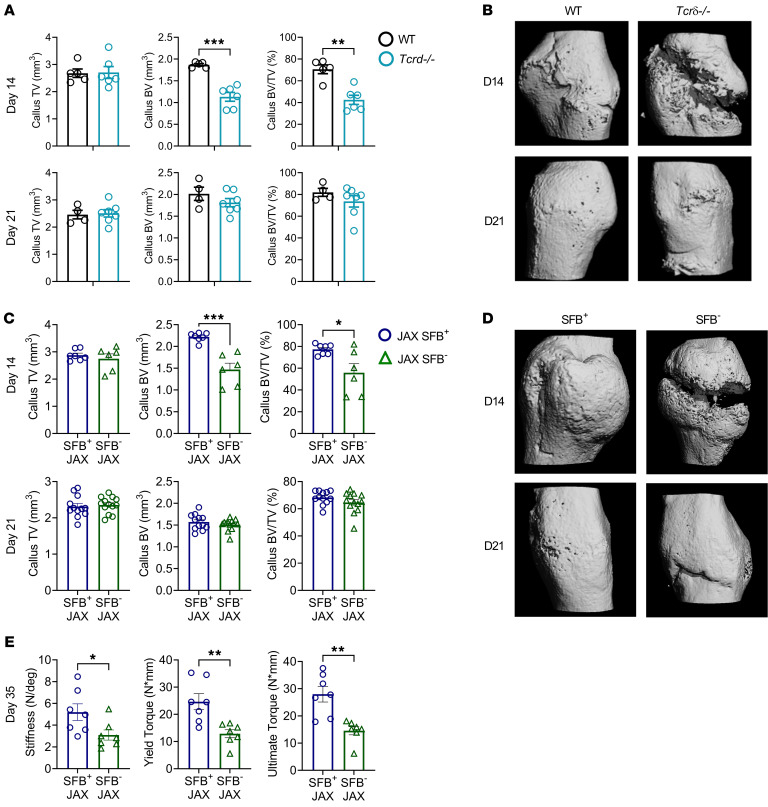
γδ T cells and Th17 cells accelerate fracture healing. Femoral fractures were induced in 12-week-old female SFB^+^ and SFB^–^ JAX mice, and SFB^+^ T*crd^–/–^* mice, a strain lacking γδ T cells. (**A**) Callus μCT measurements at days 14 and 21 PF in WT and T*crd^–/–^* mice. (**B**) Representative images of 3D μCT reconstructions of fracture callus from WT and T*crd^–/–^* mice. (**C**) Callus μCT measurements at days 14 and 21 in SFB^+^ and SFB^–^ JAX mice. (**D**) Representative images of 3D μCT reconstructions of fracture callus from SFB^+^ and SFB^–^ JAX mice at days 14 and 21. (**E**) Torsion stiffness, yield torque, and ultimate torque at day 35 PF were determined by static torsion-to-failure testing of excised femora. Data are expressed as the mean ± SEM. All data were normally distributed according to the Shapiro-Wilk normality test and analyzed by unpaired *t* test. *P < 0.05, **P < 0.01, and ***P < 0.001 compared with the indicated group. Nonsignificant comparisons are not shown.

The callus tissue surrounding the fracture site was analyzed by in vitro micro–CT (μCT). Indices indicative of callus bridging and union were measured on day 14 following fracture, a time point when the callus volume is the largest, and on day 21, a time point when consolidation and remodeling of the callus take place, leading to a reduction in callus volume. At day 14, SFB^+^ *Tcrd^–/–^* mice had a similar callus total volume at the central callus (TV_c_) but a lower bone volume (BV_c_) and callus bone volume fraction (BV_c_/TV_c_) compared with WT controls (Figure 5A). On day 21 PF, all μCT indices were similar in *Tcrd^–/–^* mice and WT controls (Figure 5A), indicating that deletion of γδ T cells impaired the early stage of fracture healing. Representative μCT images of the whole central callus confirmed impaired healing in *Tcrd^–/–^* mice as compared with WT controls at day 14 (Figure 5B). Additional studies in WT JAX mice revealed that at day 14, SFB^+^ and SFB^–^ mice had similar TV_c_. However, SFB^+^ mice had a higher BV_c_ and a higher BV_c_/TV_c_ than did SFB^–^ mice (Figure 5C). At day 21 PF, all μCT indices were similar in SFB^+^ and SFB^–^ mice (Figure 5C). Representative μCT images of the whole central callus further illustrated impaired healing in SFB^–^ mice at day 14 PF (Figure 5D). Consistent with μCT analyses, torsion stiffness, yield torque, and ultimate torque, which are indices of the force required to damage or break the healing fracture callus, were higher in SFB^+^ mice than in SFB^–^ mice at day 35 PF (Figure 5E). These findings suggest that SFB-driven expansion of Th17 cells during the early inflammatory phase leads to improved mechanical integrity of healing fractures during callus remodeling after callus union has occurred.

### Blockade of Th17 cell egress from the gut or inhibition of Th17 cell homing to the callus impairs fracture healing.

**Figure 6:**
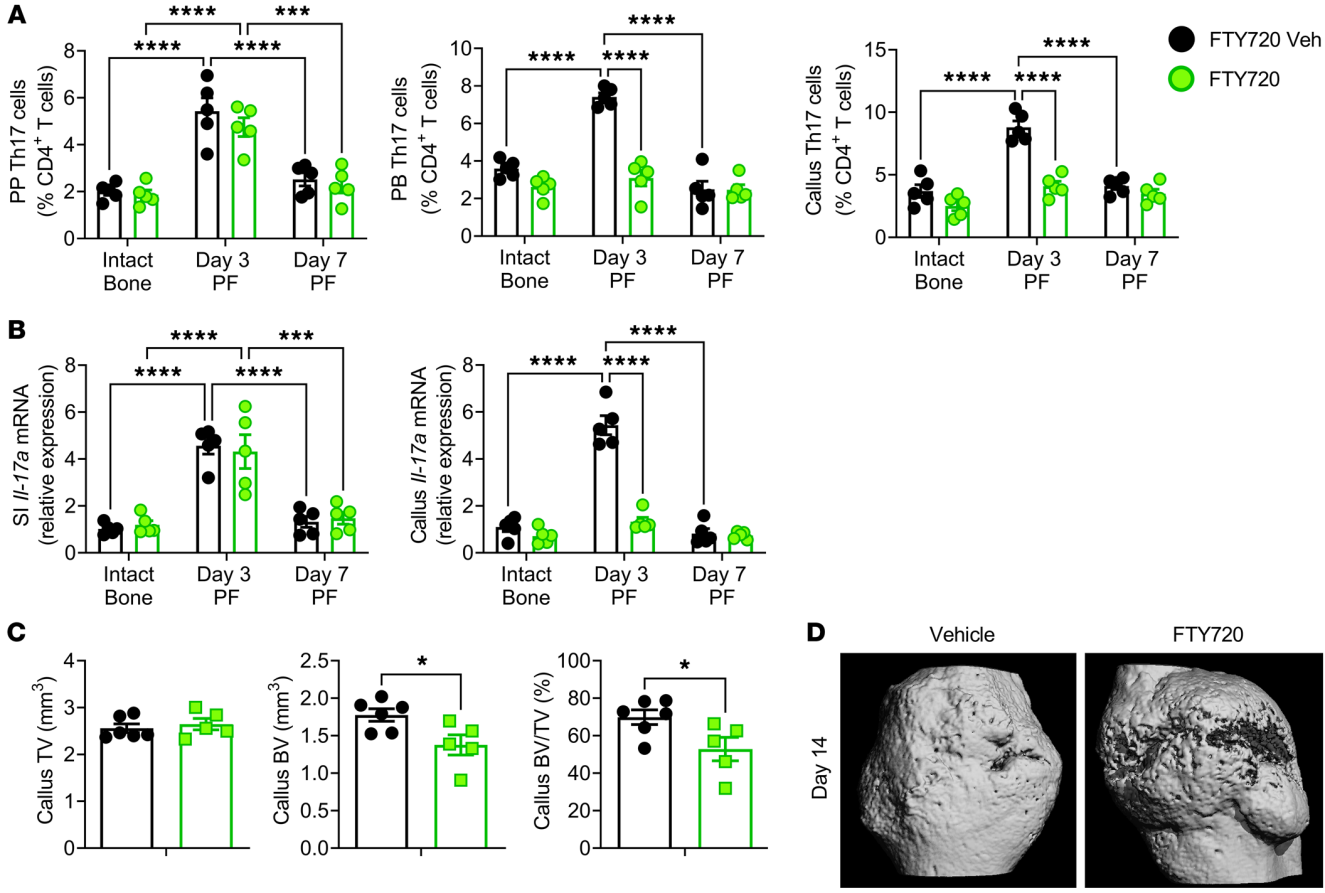
Blockade of Th17 cell egress from the intestine prevents the fracture-induced increase in peripheral blood and callus Th17 cells and impairs fracture healing. (**A**) Relative frequency of PP, peripheral blood (PB), and callus Th17 cells at days 3 and 7 PF in mice treated with the S1PR1 blocker FTY720. (**B**) Effects of fractures on the transcript levels of *Il17a* in the SI and callus in mice treated with FTY720. (**C** and **D**) Callus μCT measurements at day 14 PF. Mice were treated with FTY720 for 2 weeks starting 1 week before fracture surgery. *n* = 5–6 mice/group. Data are expressed as the mean ± SEM. All data were normally distributed according to the Shapiro-Wilk normality test and analyzed by 2-way ANOVA with post hoc Bonferroni correction for multiple comparisons, or by unpaired, 2-tailed *t* tests. *P < 0.05, ***P < 0.001, and ****P < 0.0001, compared with the indicated group. Nonsignificant comparisons are not shown.

**Figure 7:**
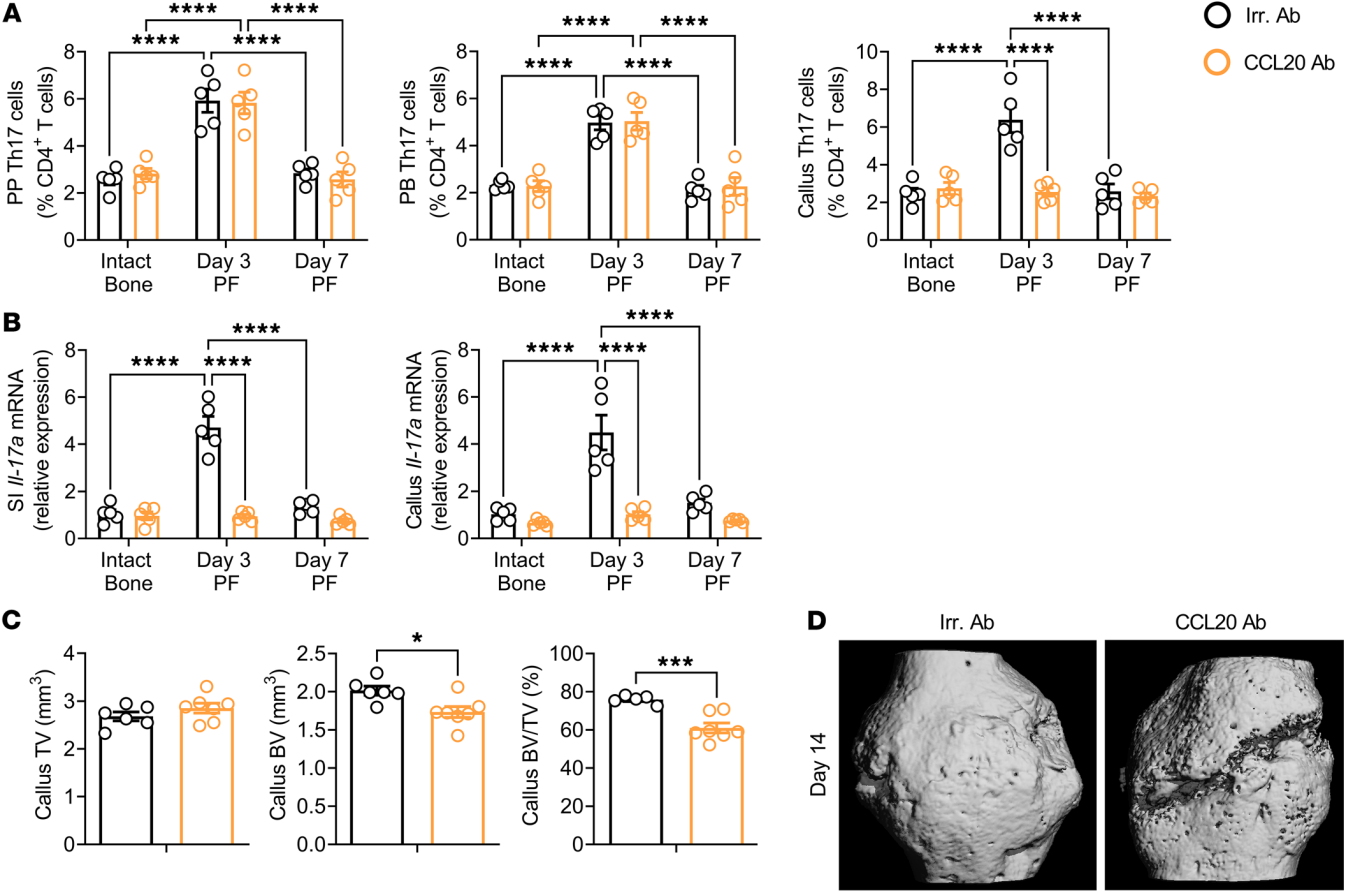
Blockade of Th17 cell influx into callus by treatment with anti CCL20 Ab prevents the fracture-induced increase in peripheral blood and callus Th17 cells and impairs fracture healing. (**A**) Relative frequency of PP, peripheral blood, and callus Th17 cells at days 3 and 7 PF in mice treated with anti-CCL20 Ab. (**B**) Effect of fractures on transcript levels of *Il17a* in the SI and callus in mice treated with anti-CCL20 Ab. (**C** and **D**) Callus μCT measurements at day 14 PF. Mice were treated with anti-CCL20 Ab or irrelevant (Irr.) Ab 1 day before surgery and every other day for 7 days. *n* = 5–6 mice/group. Data are expressed as the mean ± SEM. All data were normally distributed according to the Shapiro-Wilk normality test and analyzed by 2-way ANOVA with post hoc Bonferroni correction for multiple comparisons. *P < 0.05, ***P < 0.001, and ****P < 0.0001, compared with the indicated group. Nonsignificant comparisons are not shown.

S1PR1, a chemokine receptor expressed by T cells (32), promotes the egress of T cells from intestinal lymphoid tissues in response to the sensing of circulating S1P (32). To determine whether fractures promote the egress of Th17 cells from the intestine through a S1PR1-mediated mechanism, and to ascertain the relevance of intestinal T cell migration to the callus for fracture healing, TAC SFB^+^ mice were treated with FTY720, a SIP1R1 modulator that arrests the exit of T cells from the intestine without affecting T cell function (47, 48). FTY720 treatment was started 1 week before the induction of fractures and continued until day 7 PF. We found that FTY720 treatment did not alter the frequency of intestinal Th17 cells (Figure 6A). However, FTY720 prevented the fracture-induced increase in Th17 cells in peripheral blood and callus (Figure 6A). Moreover, FTY720 did not affect SI *Il17a* transcript levels, but decreased *Il17a* transcript levels in the callus at day 3 PF (Figure 6B). Attesting to the functional relevance of T cell trafficking, FTY720 decreased BV_c_ and BV_c_/TV_c_ at day 14 PF (Figure 6C). Representative μCT images of the whole central callus confirmed impaired healing in mice treated with FTY720 as compared with controls at day 14 PF (Figure 6D). Following their exit from the intestine, Th17 cells migrate to sites of inflammation guided by the CCR6/CCL20 axis (49). To determine the role of the CCL20-driven influx of Th17 cells into the callus, TAC SFB^+^ mice were subjected to fractures and treated with a neutralizing anti-CCL20 Ab or isotype-matched irrelevant Ab for 8 days, starting 1 day before fracture induction. Anti-CCL20 Ab did not alter the frequency of Th17 cells in PPs or peripheral blood, but it prevented the increase in callus Th17 cells induced by fractures (Figure 7A). Treatment with anti-CCL20 Ab blunted the fracture-induced increase in *Il17a* cytokine transcript levels in the SI and callus (Figure 7B). Demonstrating the relevance of CCL20-dependent migration of Th17 cells to the callus for fracture repair, treatment with anti-CCL20 Ab decreased callus BV_c_ and BV_c_/TV_c_ at day 14 (Figure 7C). Representative μCT images of the whole central callus confirmed impaired healing in mice treated with anti-CCL20 Ab as compared with controls at day 14 (Figure 7D). Together, these data support the conclusion that blockade of Th17 cell egress from the gut or inhibition of Th17 cell homing to the callus impairs fracture healing.

## Discussion

We report that γδ T cells and Th17 cells were required for optimal femoral fracture repair and that the Th17 cells implicated in fracture repair originated in the gut and then homed to the callus. We also provide evidence of trafficking of intestinal γδ T cells to the BM, a phenomenon that was not increased by fractures. The fracture-induced expansion of γδ T cells in the callus was not associated with quantitative changes in intestinal γδ T cells and preceded the expansion of intestinal and callus Th17 cells. γδ T cells were required to increase gut permeability, which in concert with an SFB-containing microbiome, promoted expansion of intestinal Th17 cells. We further report that Th17 cells egressed the intestine through a S1PR1-dependent mechanism and were guided to the callus by CCL20, a chemokine ligand expressed by callus SCs.

Reports have shown that T cells are selectively recruited to fracture sites during the early phase of repair (50) and remain at the injury site until the late phases of the healing process (51). Accordingly, their number is disproportionately elevated in the fracture site, as compared with nonfractured areas. Attesting to the beneficial role of T cells, fracture healing is delayed in patients treated with immunosuppressants, and the incidence of nonunion is higher in patients with HIV than in healthy individuals (52–54). While animal studies have confirmed that T cells contribute to fracture healing (55, 56), investigations with animals lacking both T cells and B cells showed positive or negative effects of lymphocytes on fracture healing (5, 57). The reason for this discrepancy remains undetermined. However, since B cells regulate T cells via antigen presentation and the CD40/CD40L system, it is possible that T cells may benefit fracture healing only in the presence of B cells. Few studies have analyzed the effects of specific populations of T cells in fracture healing. Effector memory CD8^+^ T cells negatively effect bone fracture healing in humans (58), but by contrast, fracture healing is improved by adoptive Treg therapy (59).

In this study, we analyzed the role of γδ T cells and Th17 cells, 2 major sources of IL-17, a cytokine critical for the inflammatory phase of fracture repair. γδ T cells are a major T cell population within the intestine (11, 12) and the musculoskeletal tissues (6). Trafficking of intestinal γδ T cells to secondary lymphoid organs had not been observed in previous studies (60). Using Kaede mice (44), we found evidence of homing of intestinal γδ T cells to the BM. However, γδ T cell migration was not increased by fractures, suggesting that the majority of γδ T cells implicated in fracture repair originate locally. γδ T cells were previously found to facilitate the repair of drilled-hole fractures (6), an observation extended by our study that examined fractures repaired by endochondral bone formation. Our findings confirmed that γδ T cells provided a prompt immune response to tissue damage by secreting IL-17 (6). This cytokine is also secreted at low levels by innate lymphoid cells, NK cells, NKT cells, neutrophils, and eosinophils (61–63), but the contribution of these cell populations to fracture repair remains unknown.

Direct effects of antigen recognition by T cells contribute to the recruitment, retention, and expansion of T cells at antigen-bearing sites (64, 65). Thus, Abx may contribute to the regulation of specific T cell populations by hampering bacterial antigen–T cell interactions. Accordingly, many populations of αβ T cells differentiate and expand in the gut and then migrate to secondary lymphoid organs or sites of inflammation. Among them are Th17 cells. Under homeostatic conditions, Th17 cells are most abundant in the gut, where their induction and accumulation are maximized by the presence of SFB. Germ-free mice, conventionally raised mice lacking SFB, and Abx-treated mice have fewer intestinal Th17 cells and a lower propensity to develop extraintestinal autoimmune disorders (34, 66). Th17 cells play a pivotal role in the bone loss resulting from pathologic conditions such as psoriasis, rheumatoid arthritis, periodontal disease, and inflammatory bowel disease (17, 67). In addition, Th17 cells have been implicated in the bone loss induced by PTH (33, 68) and estrogen deficiency (69–73). In this study, we found fractures to cause a transitory early expansion of intestinal and callus Th17 cells. While most of the Th17 cells involved were memory T cells, fractures also caused an increase in effector Th17 cells.

To directly investigate the effect of fractures on the trafficking of Th17 cells, we used Kaede mice (44). This and similar strains have been successfully used to track the migration of intestinal Th17 cells to the BM (31), kidney (35), mesenteric lymph nodes (74), and brain (75). Corroborating information was provided by injecting i.v. EGFP-Th17 cells and measuring their homing to the callus. These experiments revealed that fractures increased the tropism of Th17 cells for the callus in a TNF-dependent fashion. This is relevant because TNF is another cytokine that is expressed in the fracture site early after injury and facilitates fracture repair (76, 77).

A mechanistic finding of this study is that the egress of Th17 cells from the SI was dependent on S1PR1. This insight was obtained using FTY720, a S1PR1 modulator that arrests the exit of all lymphocytes from the intestine to the systemic circulation (47, 48). This drug is FDA approved for the treatment of multiple sclerosis. The influx of Th17 cells into the callus was mediated by the chemokine ligand CCL20, which binds to the CCR6 receptor expressed by Th17 cells (37, 46). The CCR6/CCL20 axis also drives the recruitment of intestinal Th17 cells to inflamed kidney tissue (35), and the migration of intestinal Th17 cells to the BM is induced by estrogen deficiency (31) and PTH (33).

One strength of this study was the use of the clinically relevant and widely used high-energy femoral fracture method originally developed by Bonnarens and Einhorn in the rat and later adapted to mice (78–80). These experimental fractures heal via the same endochondral ossification processes as fracture repair in humans (78). The fractures created by this method are similar in anatomical site, etiology, and fixation method to common human closed, long bone fractures resulting from traumatic events (79). These similarities may improve the translational relevance of our findings in mice. This method is also more reproducible than torsional induction of fractures and avoids confounding effects of soft tissue trauma in surgically induced fracture models (79). Stabilization of the fracture was achieved by inserting an intramedullary pin prior to fracture to maintain axial alignment during the fracture and avoid large displacements. Although stabilization devices such as nails or screws offer greater axial and rotational stability, the use of an intramedullary pin provides a sufficient stabilization for consistent healing outcomes without producing additional defects in the adjacent bone tissue associated with external fixation that may also influence repair at the fracture site.

We found that expansion of callus Th17 cells accelerated fracture repair and improved mechanical strength. It is noteworthy that the BV_c_ and BV_c_/TV_c_ were significantly higher (Figure 5) in SFB^+^ than in SFB^–^ mice only at day 14. This is consistent with a pivotal effect of Th17 cells on the sterile inflammation process that immediately precedes, and is required for, the soft callus hypertrophy, chondrogenesis, and cartilage mineralization that lead to bridging. Interestingly, the effects of the microbiota dissipated with temporal separation from the inflammatory phase, as the BV_c_ was not different between groups at the later stages of consolidation and remodeling, which are dominated by lamellar bone formation and spatially and temporally isolated from the inflammatory tissue. However, the finding of greater yield torque and ultimate torque in SFB^+^ mice at day 35 suggests that the mechanical properties of the bridged callus, which are affected by numerous volumetric, geometric, and material properties, still bear some lingering effect of the earlier delay, which is consistent with the cascading nature of the fracture repair process.

Abx are often administered to prevent infections after trauma or after surgical fracture repair. A few reports have been published about the effects of Abx on fracture repair (81–84). These studies described negative effects of some Abx on fracture repair but did not investigate whether they interfere with fracture repair by altering the gut microbiome. Our investigation suggests that broad-spectrum Abx may negatively affect fracture repair by ablating a component of the microbiome relevant for Th17 cell generation. While in healthy mice intestinal Th17 cell expansion is specifically induced by SFB (19), approximately 20 nonvirulent gut bacterial strains are known to induce Th17 cell differentiation in humans (22, 23). Thus, treatment with broad-spectrum Abx might interfere with fracture repair in humans.

Gut microbiome diversity and composition are altered by a variety of factors including sex, age, menopause, diet, living environment, and medications (85). However, the relationship between gut microbiome composition and efficiency of human fracture repairs remains to be determined. In summary, we found that locally activated γδ T cells and microbiome-dependent intestinal T cells migrating to the callus were critical for optimal fracture repair. Modifications of microbiome composition via Th17 cell–modulating bacteriotherapy and avoidance of broad-spectrum Abx may represent novel therapeutic strategies to improve fracture healing.

## Methods

### Mice.

SFB^+^ C57BL/6 (B6NTac) mice were purchased from Taconic Biosciences. SFB^–^ C57BL/6 mice (JAX: 000664), *Tnf^–/–^* mice (B6.129S-Tnf<tm1Gkl>/J, MGI:JAX 005540), *Il17a*-EGFP–knockin mice (C57BL/6-il17a tm1Bcgen/J, MGI:AX:018472), and *Tcrd^–/–^* mice (B6.129P2-Tcrd<tm1Mom/J, MGI:JAX 002120) were purchased from The Jackson Laboratory. Kaede mice [B6.Cg-c/c Tg(CAG-tdKaede)15Utr, MGI:5444247] were purchased from the RIKEN Bioresource Research Center. SFB^+^ JAX mice, SFB^+^ *Tcrd^–/–^* mice, SFB^+^ *Tnf^–/–^* mice, and SFB^+^ Kaede mice were generated by oral gavaging SFB^–^ mice with a liquid suspension of fecal pellets collected from SFB monoassociated mice, as previously described (43). SFB positivity was verified by fecal DNA extraction using the QIAamp DNA Stool Mini Kit (QIAGEN) and subsequent quantitative PCR (qPCR) using established a protocol that involving primers that are specific for the SFB 16S rRNA gene: 5′-GACGCTGAGGCATGAGAGCAT-3′ (forward) and 5′-GACGGCACGGATTGTTATTCA-3′ (reverse) and total bacterial 16S rRNA: 5′-GTGCCAGCMGCCGCGGTAA-3′ (forward) and 5′-GGACTACHVGGGTWTCTAAT-3′ (reverse). (86). All experiments were conducted using female mice. All mice entering Emory University were shipped to the same room within the same vivarium at the Whitehead Biomedical Research Building. All mice were housed under specific pathogen–free conditions and were fed γ-irradiated 5V5R mouse chow (Purina Mills) and autoclaved water ad libitum. The animal facility was kept at 23 °C (±1°C) with 50% relative humidity and a 12-hour light/12-hour dark cycle. All mice were acclimatized within our facility for at least 3 days before experimentation.

### Fracture surgery.

A well-established closed femoral fracture model (78, 80, 87) was used for our studies. Briefly, after opening the skin and the knee joint of the mouse, the patella was slid to the medial side of the condyle and the condyle was exposed by flexing the knee. A 25 gauge needle was inserted into the intramedullary canal to create an initial opening, and then the needle was removed. A 25 gauge spinal needle (EXEL Int) was inserted into the opening and down the length of the medullary canal until meeting the cortical bone of the greater trochanter of the proximal femur. The tip of the pin was then buried under the surface of the condyle. The length of the pin was further trimmed using a wire cutter. The incision was then closed with 5-0 absorbable suture. Following the surgical procedure, the fracture was generated by dropping a weight onto the operated extremity. Immediately PF and before the animal revived from anesthesia, an x-ray was taken with a Kubtec DIGImus digital cabinet to check that placement of the intramedullary pin was adequate and that the fracture was mid-diaphyseal without comminution. Animals were excluded from the study if their fractures were misaligned, heavily comminuted, or poorly reduced, or if the pins appeared bent in the x-ray or μCT images.

### Depletion of gut commensal microflora.

Cocktails of broad-spectrum Abx (1 mg/mL ampicillin, 0.5 mg/mL vancomycin, 1 mg/mL neomycin sulfate, and 1 mg/mL metronidazole benzoate/metronidazole) were prepared as described by Rakoff-Nahoum et al. (88), or of nonabsorbable Abx (2 mg/mL bacitracin and 2 mg/mL neomycin sulfate) were included in the drinking water of mice starting 1 week before the initiation of fractures. Abx water was prepared freshly and changed twice a week until sacrifice. Fecal microbiome depletion was verified by fecal DNA extraction using the QIAamp DNA Stool Mini Kit (QIAGEN) and subsequent qPCR following an established protocol that used primers specific for the detection of the 16S rRNA gene present in all bacteria (5′-GTGCCAGCMGCCGCGGTAA-3′, forward; 5′-GGACTACHVGGGTWTCTAAT-3′, reverse), as previously described (33, 89).

### Kaede mouse cell photoconversion.

Kaede mice express a photoconvertible fluorescence protein that changes from green (518 nm) to red (582 nm) upon exposure to near-UV (350–410 nm) light. Twelve-week-old male SFB^+^ Kaede mice were subjected to fracture surgery. Two days after surgery, all animals underwent surgical laparotomy, during which the caecum and distal SI were eviscerated, and the 4 SI PPs most proximal to the caecum were identified and illuminated with 390 nm wavelength light for 2 minutes each. The caecum and distal SI were reinserted into the abdominal cavity, and the abdominal wall was closed. Aluminum foil was used to protect tissue other than target PPs from light during exposure. Twenty-four hours after photoconversion, mice were sacrificed, and PP cells were collected. A single-cell suspension was prepared and analyzed by flow cytometry. Callus cells were also collected at sacrifice and single-cell suspension was prepared. Callus KaedeR total T cells were enumerated by flow cytometry by analyzing whole callus cells. Since the number of callus Th17 cells is low, callus cells were enriched for T cells or CD4^+^ T cells by positive immunomagnetic sorting using mouse CD3ε MicroBeads (Miltenyi Biotec) or mouse CD4 (L3T4) MicroBeads (Miltenyi Biotec). These enriched cell populations were then used for intracellular staining and flow cytometric enumeration of callus Th17 cells.

### EGFP^+^ Th17 cell transfer.

*Il17a*-EGFP–knockin mice (C57BL/6-IL17atm1Bcgen/J) express EGFP as a marker of IL-17A activity. Naive CD4^+^ T cells (CD4^+^CD44^lo^CD62L^hi^ cells) were isolated from the spleens of *Il17a*-EGFP mice using the EasySep Mouse Naive CD4^+^ T Cell Isolation Kit (STEMCELL Technologies). EGFP^–^ naive CD4^+^ T cells were cultured in Th17-polarizing conditions for 4 days using the Mouse Th17 Cell Differentiation Kit (R&D Systems) to generate EGFP^+^CD4^+^ Th17 cells. EGFP^+^CD4^+^ live Th17 cells were FACS sorted by a FACSAria II (BD Biosciences) and injected i.v. (1 × 10^6^ cells per mouse) into SFB^–^ JAX WT and *Tnf^–/–^* recipient mice, which had been subjected to fractures 2 days before the T cell transfer. One day after transfer, the relative and absolute frequencies of EGFP^+^CD4^+^ T cells in the callus of recipient mice were determined by flow cytometry.

### Preparation of PP and callus tissue single-cell suspensions.

For PP cell isolation, the SI was removed and flushed of fecal content. PPs were excised and collected in 1 mL cooled RPMI 1640. PPs were dissociated using the plunger of a 2.5 mL syringe and gently forced through a 70 μm cell strainer placed over a 50 mL tube. A single-cell suspension was used for flow cytometric analysis. For callus cell isolation, callus tissues were sectioned into a few small pieces and digested with a collagenase/dispase solution (MilliporeSigma) at a concentration of 1 mg/mL dissolved in RPMI medium (Gibco, Thermo Fisher Scientific) for 1 hour at 37°C. Debris was removed by sterile filtration with 70 μm strainers (BD Bioscience), followed by the addition of RBC lysis buffer (BioLegend) to remove erythrocytes. The resulting single-cell suspension was used for flow cytometric analysis.

### FTY720 treatment.

The S1PR1 functional antagonist FTY720 (2-amino-2-[2-(4-octylphenyl) ethyl]propane-1,3-diol) (MilliporeSigma) was added to the drinking water at 5 μg/mL as described by Krebs et al. (36). FTY720 treatment was initiated 1 week before fracture surgery and continued for 1 week after the surgery. Water containing FTY720 was changed weekly.

### In vivo anti-CCL20 Ab treatment.

Mouse anti-CCL20 Abs (clone 114908, R&D Systems) or an isotype control (clone 43414, R&D Systems) were injected i.p. at 50 μg per mouse 1 day before fracture surgery and every other day thereafter for 7 days.

### μCT measurements.

Bone volume and structure of the whole callus volume or a “shell” of the callus perimeter centered at the fracture site were evaluated ex vivo by μCT using a Scanco μCT50 as previously published (90–93). Transverse tomographic images of the entire callus were acquired. To analyze the entire central callus, a tight contour of the outer perimeter of the callus was created in 100 slices (1.6 mm) centered on the fracture line using a semiautomated algorithm (91), and host cortical bone was excluded by manual contouring. For analysis of the outer callus “shell,” a 160 μm thick donut was created by peeling voxels from the perimeter contour. Mineralized tissue was segmented from soft tissue using an optimized threshold and noise filter (MilliporeSigma 1.0, support 1, threshold 76 mmHA/cm^3^), and the total volume (TV_c_), bone volume (BV_c_), and BV_c_/TV_c_ of the central callus were measured using previously published methods (94). The results of this method are highly correlated with biomechanical strength and callus bridging (91).

### Mechanical testing.

Static torsion testing was used to assess the mechanical integrity of the healing femora. Briefly, the bones were cleaned of soft tissue, and the intramedullary pin was carefully removed prior to potting both ends in copper solder cap fittings (Mueller Streamline 1/2 inch diameter, item no. 652243) with polymethyl-methacrylate (PMMA) (Ortho-Jet, Lang Dental). The torsional tests were conducted on a servohydraulic material testing system (Dynamight, Instron) with a rotary actuator. The torsional rigidity or stiffness (slope of the linear region) and ultimate torque (maximum torque value prior to fracture) were determined from T versus θ curves using a custom MATLAB script (MathWorks).

### Flow cytometry.

Flow cytometry was performed on an Aurora system (Cytek) and a BD FACSymphony A5 (BD Biosciences), and data were analyzed using FlowJo software (Tree Star). For cell-surface staining, cells were stained with anti–mouse purified CD16/-32 Ab (clone 93), anti–BV 510-CD45 Ab (clone 30-F11), anti–BV 421-TCRβ Ab (clone H57-597), anti–PerCP/Cy5.5-CD4 Ab (clone RM4-5), anti–BV 711-CD8 Ab (clone 53-6.7), Alexa Fluor 700-CD3 Ab (clone 17A2), anti–PE/Cy7-CD3ε Ab (clone KT3.1.1), anti–FITC-TCR γ/δ Ab (clone GL3), anti–BV 421-CD44 Ab (IM7), anti–APC-CD62L Ab (MEL-14), anti–PE-Cy7-CD69 Ab (H1.2F3), anti–BV605-CD127 Ab (A7R34), anti–APC-Rat IgG1 Ab, anti-κ isotype control Ab (RTK2071), anti–PE-rat IgG1 Ab, κ isotype control Ab (RTK2071), anti–FITC-Rat IgG1, κ isotype control Ab (eBRG1), APC-eFlour780-Rat IgG1, κ isotype control Ab (eBRG1), anti–PerCP/Cy5.5-F4/80 Ab (clone BM8) (all from BioLegend), and anti–FITC-Vβ T cell receptor Ab (clone 38397) (BD Biosciences). The live cells were discriminated using the Zombie NIR Fixable Viability Kit or the Zombie Aqua Fixable Viability Kit (BioLegend). For intracellular staining, cells were incubated with cell activation cocktail (BioLegend) in the presence of monensin solution at 37°C for 12 hours. Anti–mouse PE–IL-17A Ab (clone eBio17B7), anti–PE-IL-6 Ab (MP5-20F3), anti–APC-eFlour 780-IL-1β Ab (NJTEN3), anti–FITC-TNF-α Ab (MP6-XT22), and anti–APC-IL-17 Ab (TC11-18H10.1) (all from Thermo Fisher Scientific) were added after cell fixation and permeabilization with the Intracellular Fixation & Permeabilization Buffer Set (Thermo Fisher Scientific).

### qPCR and primers.

Total RNA was isolated using the RNeasy kit (QIAGEN), and cDNA was synthesized with random hexamers using Superscript II (Invitrogen, Thermo Fisher Scientific) according to the manufacturer’s instructions. The relative abundance of cDNAs was computed by qPCR analysis using the ABI StepOnePlus Real-Time PCR system (Applied Biosystems). The expression levels of murine *Il-1b*, *Il6*, *Il17a*, and *Tnf* were measured in callus cells by qPCR. Changes in relative gene expression between intact and fractured groups were calculated using the 2^–ΔΔCt^ method with normalization to 18S rRNA. The primers used are listed in Supplemental Table 1.

### RNA isolation from SI.

A 10 mm intestinal piece from each mouse was collected at sacrifice and stored at –80°C. Tissue was disrupted and homogenized using RNase-Free stainless-steel beads in the Bullet Blender Strom 24 blender (Next Advance). RNA was extracted using TRIzol reagent (Thermo Fisher Scientific) and the DNase Max kit (QIAGEN). The expression levels of murine *Il-1b*, *Il6*, *Il17a*, and *Tnf* were measured in SI cells by qPCR.

### Statistics.

All data were normally distributed according to the Shapiro-Wilk normality test. Data were analyzed using an unpaired, 2-tailed *t* test or a 1-way or 2-way ANOVA, as appropriate. This analysis included the main effects for animal strain and treatment plus the statistical interaction between animal strain and treatment. When the statistical interaction was statistically significant (*P* < 0.05) or suggestive of an important interaction, then *t* tests were used to compare the differences between the treatment means for each animal strain, applying the Bonferroni correction for multiple comparisons.

## Author contributions

RP designed the studies. HYD, SP, AS, SU, and JSN performed the research and analyzed the data. RP, DSP, MNW, and RMJ, wrote the manuscript.

## Supplementary Material

Supplemental data

Supplemental table 1

## Version 1 03/07/2023

In-Press Preview

## Version 2 04/17/2023

Electronic publication
