# Supplementary material for: Callus γδ T cells and microbe-induced intestinal Th17 cells improve fracture healing in mice
Source: J Clin Invest. 2023 Apr 17;133(8):e166577. doi: 10.1172/JCI166577 (PMC10104897; doi:10.1172/JCI166577)
Supplement: Supplemental data [file jci-133-166577-s206.pdf]

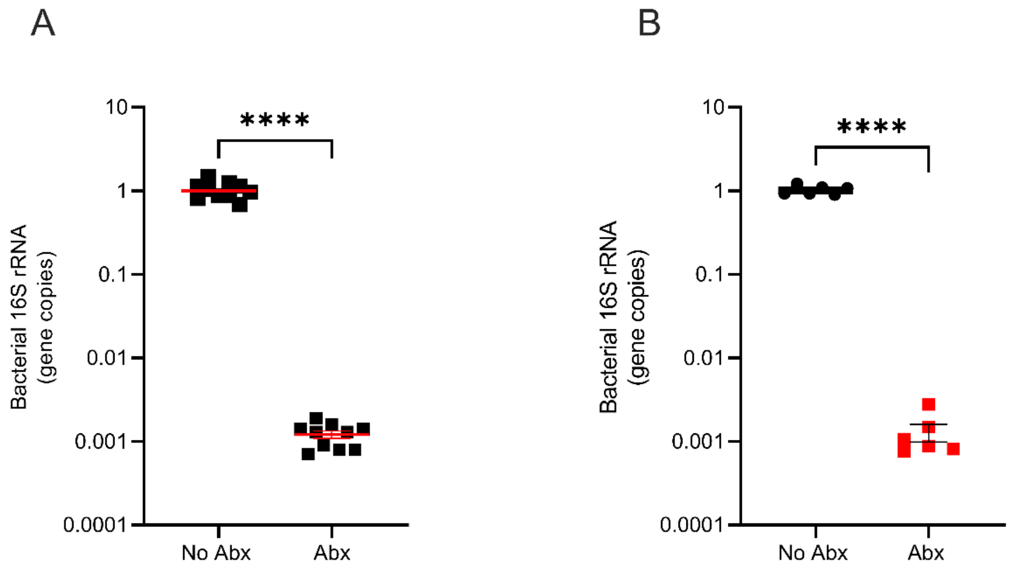

Supplementary Figure 1. Analysis of fecal bacterial-specific DNA. (**A**) Effect of broad-spectrum antibiotics (Abx). Femoral fractures were induced in 12-wk-old female SFB$^+$ JAX mice. Mice were treated with broad-spectrum antibiotics (1 mg/mL ampicillin, 0.5 mg/mL vancomycin, 1 mg/mL neomycin sulfate, 1 mg/mL metronidazole administered in drinking water) for 4 weeks, starting 1 week before fracture induction. (**B**) Effect of non-absorbable Abx. Femoral fractures were induced in 12-wk-old female SFB$^+$ TAC mice. Mice were treated with non-absorbable Abx (1 mg/mL neomycin sulfate, 1 mg/mL bacitracin dissolved in drinking water) for 4 weeks, starting 1 week before initiation of fractures. Stools were collected at day 21 post fracture. Genomic DNA was isolated from equal amounts of fecal material. Relative quantitation of 16S rRNA gene copies of total bacteria were determined by qPCR, using universal 16S rRNA primers. n = 6-10 mice per group. Data are expressed as Mean $\pm$ SEM. Data were analyzed by unpaired t-tests. ****=p<0.0001 compared to the indicated group.

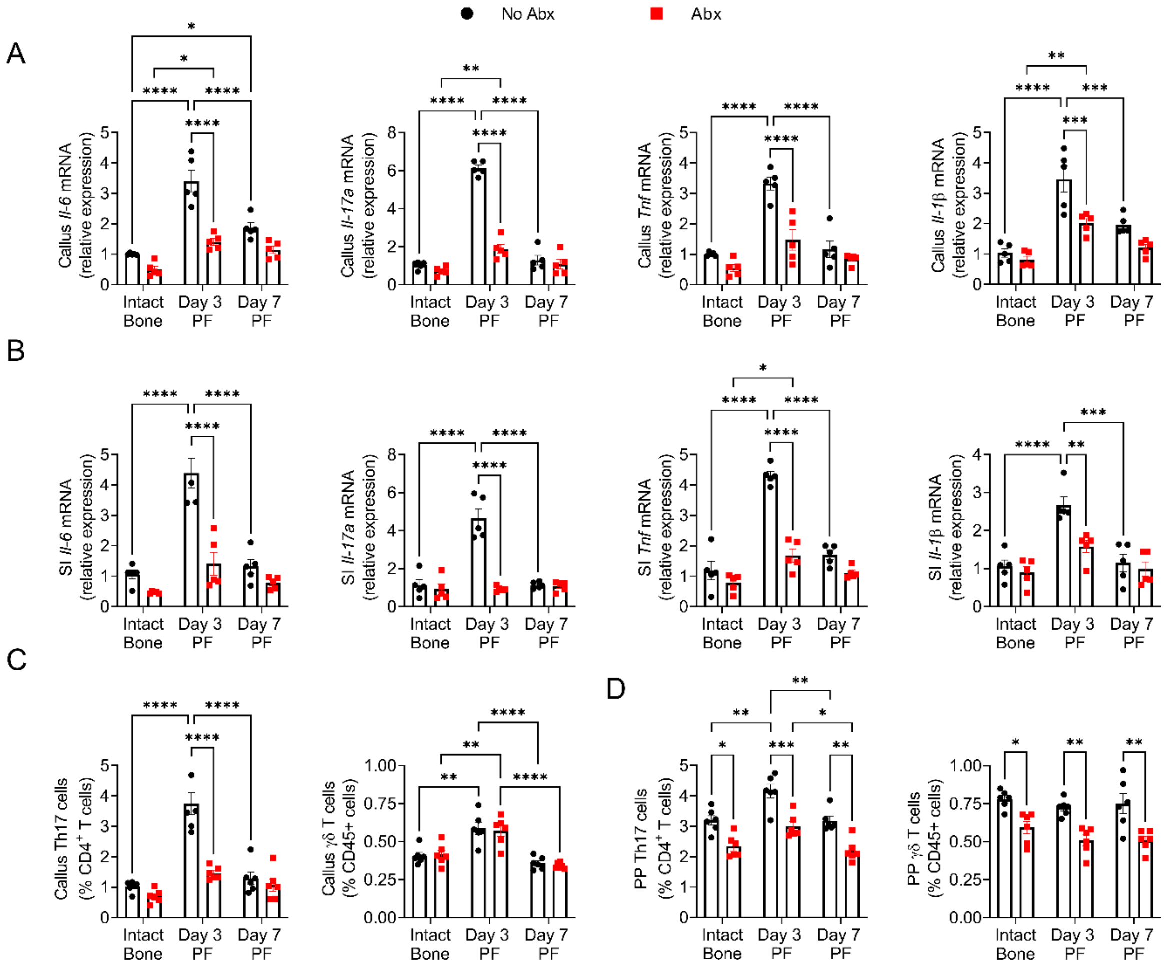

Supplementary Figure 2. Effects of microbiota depletion by non-absorbable antibiotics (Abx) on callus and small intestinal (SI) cytokine transcripts, γδ T cell and Th17 cell frequency, and fracture healing. Femoral fractures were induced in 12-wk-old female SFB$^+$ TAC mice. Mice were treated with and without non-absorbable Abx staring 1 week before fractures. (**A**) Callus levels of *Il6, Il17a*, *Tnf* and *Il1β* transcripts. (**B**) SI levels of *Il6, Il17a*, *Tnf* and *Il1β* transcripts. (**C**) Relative frequency of callus Th17 cells (TCRβ$^+$CD45$^+$CD4$^+$IL-17A$^+$ cells) and γδ T cells (CD3ε$^+$CD45$^+$TCRγδ$^+$ cells). (**D**) Relative frequency of Peyer's Patches (PPs) Th17 cells (TCRβ$^+$CD45$^+$CD4$^+$IL-17A$^+$ cells) and γδ T cells (CD3ε$^+$CD45$^+$TCRγδ$^+$ cells). PF = post fracture. n= 5 mice/group. Data are expressed as Mean ± SEM. All data were normally distributed according to the Shapiro-Wilk normality test. Data were analyzed by two-way analysis-of-variance and post hoc tests applying the Bonferroni correction for multiple comparisons, *=p<0.05, **=p<0.01, ***=p<0.001 and ****= p<0.0001 compared to the indicated group. Nonsignificant comparisons not shown.

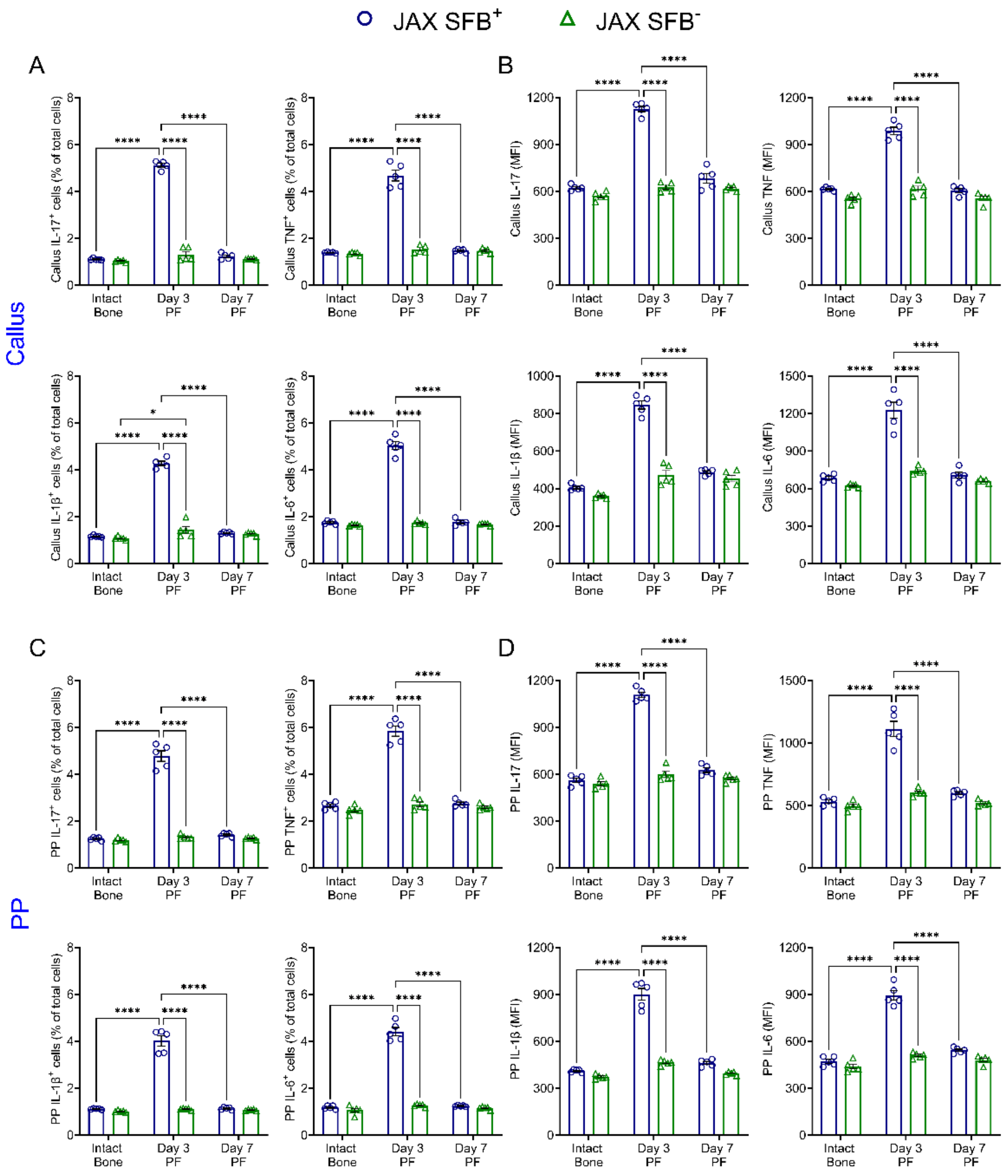

Supplementary Figure 3. Effects of fractures on the level of callus and Peyer's patches (PP) intracellular inflammatory cytokine protein levels. (**A**) Relative frequency of IL-17, TNF, IL-1β, and IL-6 positive cells in the callus of SFB^+ and SFB^- JAX mice. (**B**) MFI values for IL-17, TNF, IL-1β, and IL-6 in callus cells of SFB^+ and SFB^- JAX mice. (**C**) Relative frequency of IL-17, TNF, IL-1β, and IL-6 positive cells in PPs of SFB^+ and SFB^- JAX mice. (**D**) MFI values for IL-17, TNF, IL-1β, and IL-6 in PPs of SFB^+ and SFB^- JAX mice. Femoral fractures were induced in 12-wk-old female SFB^+ JAX and SFB^- JAX mice. Callus tissue and PPs were harvested from intact bone or at 3 and 7 days after fracture, fixed, permeabilized, stained and analyzed by flow cytometry. PF= post fracture. n= 5 mice/group. Data are expressed as Mean + SEM. All data were normally distributed according to the Shapiro-Wilk normality test and analyzed by two-way analysis-of-variance and post hoc tests applying the Bonferroni correction for multiple comparisons. ****= p<0.0001 compared to the indicated group. Nonsignificant comparisons not shown.

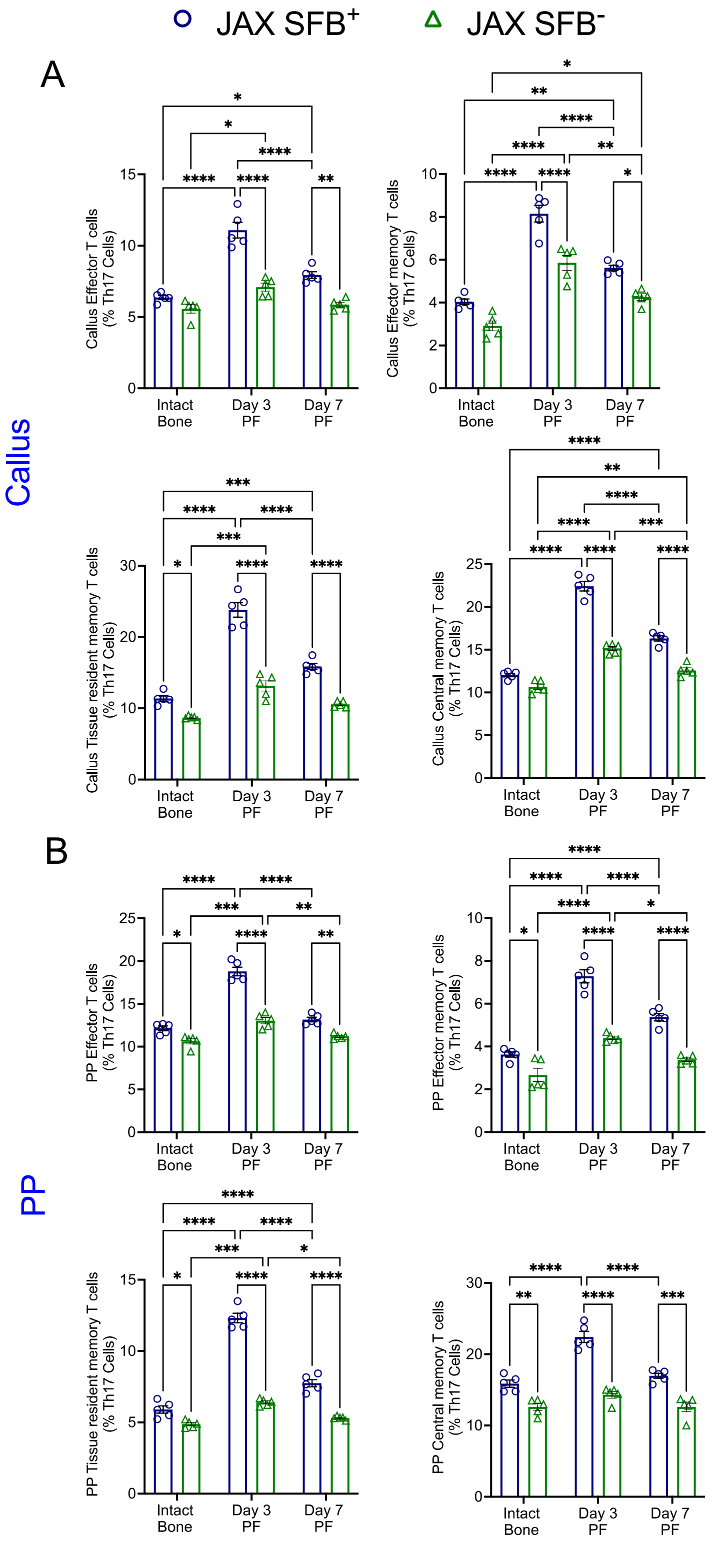

Supplementary Figure 4. Effects of fractures on Th17 cell subsets in the callus (Panel A) and PPs (Panel B). Panels show the relative frequency of effector ($CD44^+CD62L^-CD127^-$) effector memory ($CD44^+CD62L^-CD127^+$), tissue resident memory ($CD44^+CD62L^-CD69^+$), and central memory ($CD44^+CD62L^-CD127^+$) Th17 cells ($CD45^+CD3^+CD4^+$IL-17A$^+$ cells). PF= post fracture. n= 5 mice/group. Data are expressed as Mean + SEM. All data were normally distributed according to the Shapiro-Wilk normality test and analyzed by two-way analysis-of-variance and post hoc tests applying the Bonferroni correction for multiple comparisons. **=p<0.01, **=p<0.01, ***=p<0.001 and ****= p<0.0001 compared to the indicated group. Nonsignificant comparisons not shown.

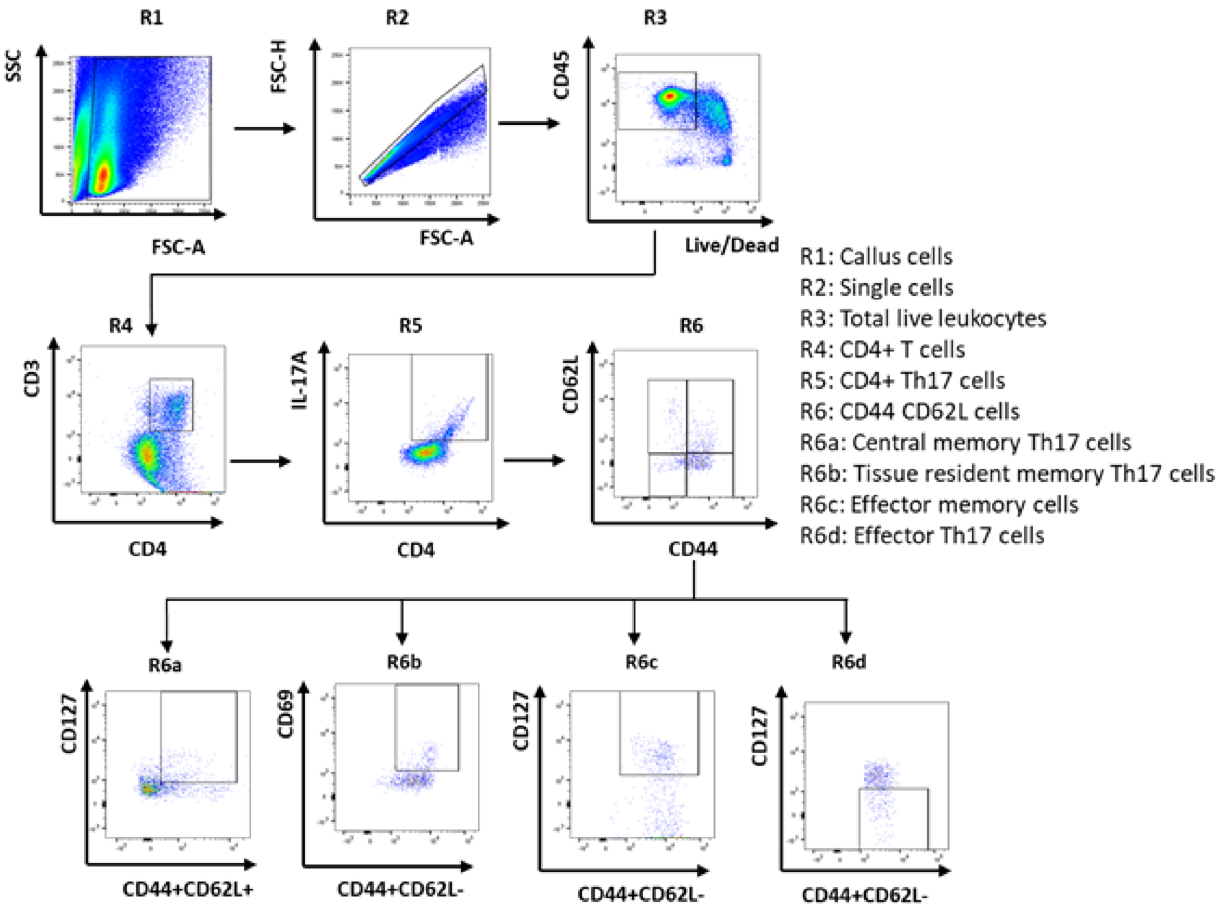

Supplementary Figure 5. Gating strategy used to identify Th17 cells, effector Th17 cells, effector memory Th17 cells, tissue resident memory Th17 cells, and central memory Th17 cells in callus and PPs. Following red blood cells lysis, single cell suspensions were prepared from callus and PPs and stained with antibodies to the indicated antigens and live/dead cell dye. Gated regions are numbered from R1 to R6d. The figure shows one representative gating of flow cytometric plots.

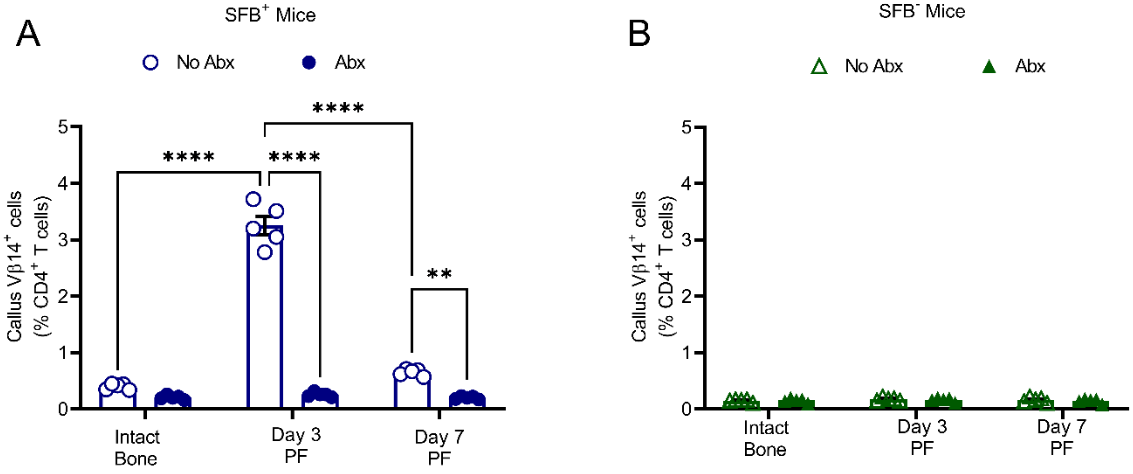

Supplementary Figure 6. Fractures increase the homing of intestinal total vβ14+ T cells to the callus via a microbiota dependent mechanism. Femoral fractures were induced in 12-wk-old female SFB^+ and SFB^- JAX mice**.** Mice were treated with and without broad-spectrum antibiotics (Abx) starting 1 week before fractures. (**A**) Relative frequency of callus $v\beta14^+$ Th17 cells in SFB^+ JAX mice. (**B**) Relative frequency of callus $v\beta14^+$ Th17 cells in SFB^- JAX mice. PF= post fracture. n= 5 mice/group. Data are expressed as Mean $\pm$ SEM. All data were normally distributed according to the Shapiro-Wilk normality test and analyzed by two-way analysis-of-variance and post hoc tests applying the Bonferroni correction for multiple comparisons. **=p<0.01, and ****= p<0.0001 compared to the indicated group. Nonsignificant comparisons not shown.

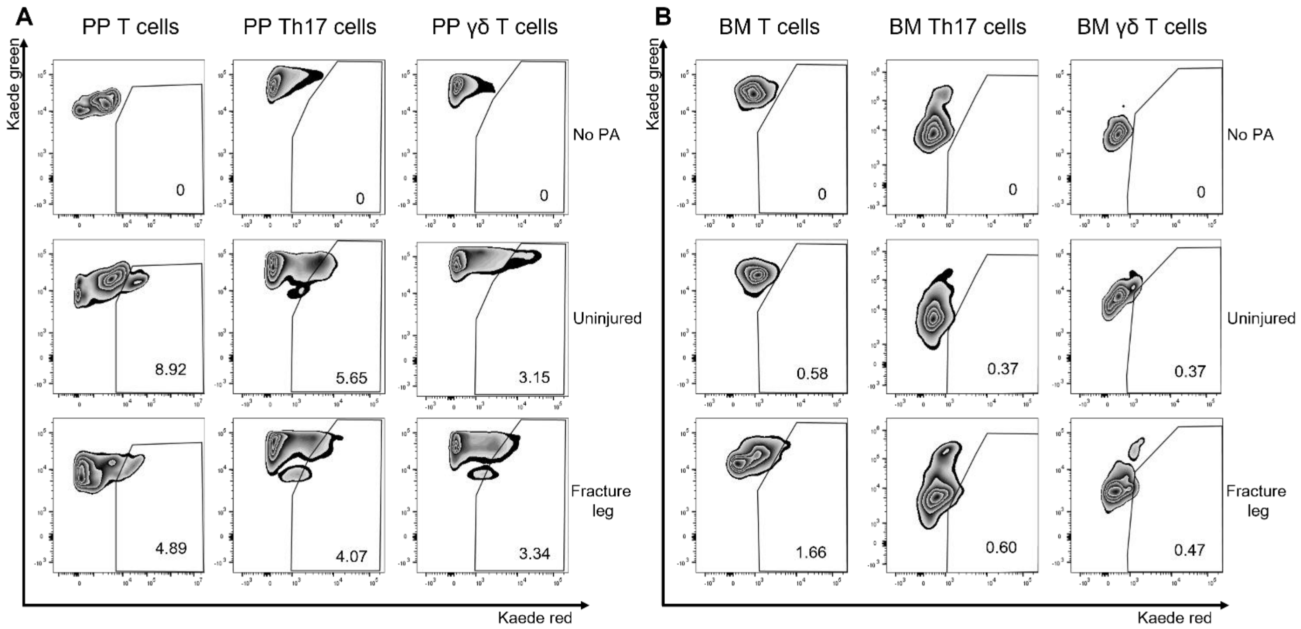

Supplementary Figure 7. Representative flow cytometric analysis of Peyer's patch (PP) cells and callus cells from Kaede mice subjected or not subjected to photoactivation (PA). Femoral fractures were induced in 12-week-old male SFB$^+$ Kaede mice. Femoral fractures were induced in 12-week-old male SFB$^+$ Kaede mice. After 2 days, 4 Peyer's patches (PPs) were surgically exposed and illuminated with a near-UV light for 2 mins. After 1 day, mice were sacrificed and the frequency of PP and callus red fluorescing ab T cells, Th17 cells and γδ T cells was determined by flow cytometry. PP red fluorescing T cells were counted in PPs from mice with fractures and PPs from uninjured control mice. Callus red fluorescing T cells were counted in the callus tissue of fractured femurs, and BM from the contralateral uninjured femur, and BM from uninjured mice. (**A**) Relative frequency of KaedeR total T cells, Th17 cells, and γδ T cells in PP. (**B**) Relative frequency of KaedeR total T cells, Th17 cells, and γδ T cells callus.
