# Supplementary material for: Callus γδ T cells and microbe-induced intestinal Th17 cells improve fracture healing in mice
Source: J Clin Invest. 2023 Apr 17;133(8):e166577. doi: 10.1172/JCI166577 (PMC10104897; doi:10.1172/JCI166577)
Supplement: Supplemental table 1 [file jci-133-166577-s207.pdf]

Supplemental Table 1. Primer sequences for real-time PCRs.

	Forward primer sequence (5'-3')	Reverse primer sequence (5'-3')
**Mouse TNF**	AACTCCAGGCGGTGCCTAT	TGCCACAAGCAGGAATGAGA
**Mouse IL-17A**	TGACGCCCACCTACAACATC	CATCATGCAGTTCCGTCAGC
**Mouse IL-1β**	TTCAGGCAGGCAGTATCACTC	GAAGGTCCACGGGAAAGACAC
**Mouse IL-6**	TAGTCCTTCCTACCCCAATTTCC	TTGGTCCTTAGCCACTCCTTC
**18s ribosomal RNA**	ATTCGAACGTCTGCCCTATCA	GTCACCCGTGGTCACCATG
**Total bacterial 16S rRNA**	515F (GTGCCAGCMGCCGCGGTAA)	806R (GGACTACHVGGGTWTCTAAT)
**SFB 16S rRNA**	736F (GACGCTGAGGCATGAGAGCAT)	844R (GACGGCACGGATTGTTATTCA)
